# Microbial inoculants with higher capacity to colonize soils improved wheat drought tolerance

**DOI:** 10.1111/1751-7915.14350

**Published:** 2023-10-10

**Authors:** Jiayu Li, Juntao Wang, Hongwei Liu, Catriona A. Macdonald, Brajesh K. Singh

**Affiliations:** ^1^ Hawkesbury Institute for the Environment, Western Sydney University New South Wales Penrith Australia; ^2^ Global Centre for Land‐Based Innovation Western Sydney University New South Wales Penrith Australia; ^3^ School of Science Western Sydney University Penrith New South Wales Australia

## Abstract

Microbial inoculants have gained increasing attention worldwide as an eco‐friendly solution for improving agriculture productivity. Several studies have demonstrated their potential benefits, such as enhanced resistance to drought, salinity, and pathogens. However, the beneficial impacts of inoculants remain inconsistent. This variability is attributed to limited knowledge of the mechanisms by which microbial inoculants affect crop growth and a lack of ecological characteristics of these inoculants that limit our ability to predict their beneficial effects. The first important step is believed to be the evaluation of the inoculant's ability to colonize new habitats (soils and plant roots), which could provide crops with beneficial functions and improve the consistency and efficiency of the inoculants. In this study, we aimed to investigate the impact of three microbial inoculants (two bacterial: P1 and P2, and one fungal: P3) on the growth and stress responses of three wheat varieties in two different soil types under drought conditions. Furthermore, we investigated the impact of microbial inoculants on soil microbial communities. Plant biomass and traits were measured, and high‐throughput sequencing was used to characterize bulk and rhizosphere soil microbiomes after exposure to drought stress. Under drought conditions, plant shoot weight significantly increased (11.37%) under P1 treatments compared to uninoculated controls. In addition, total nitrogen enzyme activity increased significantly under P1 in sandy soil but not in clay soil. Importantly, network analyses revealed that P1, consisting of *Bacillus paralicheniformis* and *Bacillus subtilis*, emerged as the keystone taxa in sandy soil. Conversely, P2 and P3 failed to establish as keystone taxa, which may explain their insignificant impact on wheat performance under drought conditions. In conclusion, our study emphasizes the importance of effective colonization by microbial inoculants in promoting crop growth under drought conditions. Our findings support the development of microbial inoculants that robustly colonize plant roots for improved agricultural productivity.

## Graphical abstract

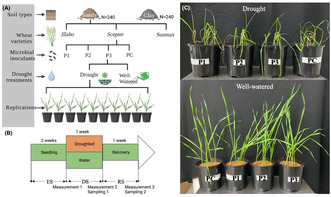

## INTRODUCTION

Drought is one of the most dramatic abiotic factors impeding the growth of global grain productivity (Lesk et al., 2022). With climate change scenarios predicting an increase in drought events across many regions worldwide, the urgency of addressing this issue is paramount. Drought stress directly affects the physiological activities of plants by decreasing their photosynthetic and transpiration rates (Joshi et al., 2022). Additionally, it stimulates stress ethylene synthesis and the generation of reactive oxygen species within plants, resulting in the degradation of proteins, enzyme activity, and disruption of DNA strands, and consequently inhibits the growth of both plant roots and shoots as well as crop yield and quality (Das & Roychoudhury, 2014; Hasanuzzaman et al., 2021; Naghizadeh et al., 2019). Drought can also have indirect negative impacts on crop growth by disturbing soil microbial communities, limiting their nutrient uptake, and compromising crucial soil functions essential for sustaining plant growth and health. For example, drought can negatively impact microbial communities by reducing microbial diversity (Preece et al., 2019) and disrupting microbial networks (de Vries et al., 2018), a scenario of concern for plants and health because these communities regulate soil biogeochemical processes and nutrient transformations, acting as significant indices for assessing soil health and fertility (Di Salvo et al., 2018; Fierer et al., 2021; Nazaries et al., 2013).

Plant growth‐promoting microbes (PGPMs) provide a sustainable solution to alleviate the impact of abiotic stresses on crops (Elnahal et al., 2022; Li et al., 2022; Souza et al., 2015). Importantly, they also contribute positively to overall ecosystem health, supporting beneficial organisms such as pollinators via reduced demands for synthetic fertilizers/insecticides in the agriculture sector. Root‐associated microbes from genera including *Azospirillum*, *Arthrobacter*, *Azotobacter*, *Bacillus*, *Bradyrhizobium*, *Erwinia*, *Enterobacter*, *Klebsiella*, *Nitrobacter*, *Paenibacillus*, *Pantoea*, *Pseudomonas*, *Rhizobium*, *Serratia*, and *Xanthomonas* were reported as PGPMs that can be potentially developed into microbial inoculants (Di Benedetto et al., 2017; Hayat et al., 2010). Some of them can improve the drought tolerance of plants. For example, the microbial inoculant strains IG 3 (*Klebsiella* sp.), IG 10 (*Enterobacter ludwigii*), and IG 15 (*Flavobacterium* sp.) have been reported to improve wheat drought tolerance at the seedling stage (Gontia‐Mishra et al., 2016). Further, the two bacteria, *Enterobacter cloacae* and *Citrobacter* sp., were reported to significantly enhance wheat biomass under salinity and drought conditions (Gontia‐Mishra et al., 2017). However, the effectiveness of such inoculants often yields inconsistent results due to various factors. These include the competitive abilities of the microbial inoculants, their capacity to colonize different niches and cultivars of plant species, as well as environmental conditions such as temperature, moisture levels, and soil pH (Lopes et al., 2021; O'Callaghan et al., 2022). Microbial inoculants are introduced to soil ecosystems, where they interact with the indigenous microbial communities that are already present. Their ability to outcompete the native microflora and colonize plant roots is crucial for successful outcomes. In this context, different plant species/varieties may have varying abilities to support different microbial inoculants, and the effectiveness of microbial inoculants on varieties may vary (Santos et al., 2019). Most importantly, changing environmental factors can have a significant impact on the efficacy of microbial inoculants due to their influence on the survival and activity of the introduced microbial inoculants as well as the indigenous microbial communities. Despite this conceptual knowledge, empirical evidence is lacking on how microbial inoculants contribute to crop plant drought tolerance and what factors determine the inoculant's ability to provide target functions.

Microbial inoculants require to invade, establish, proliferate, and likely drift and these steps together determine their impact on plant performance interaction with indigenous soil microbes (Kang et al., 2013; Singh et al., 2023). Given these, it is proposed that incorporating an ecological framework in evaluating and applying microbial products can potentially predict colonization success and thus the benificial outcomes of inoculants (Singh et al., 2023). In addition, keystone taxa (microorganisms that play a critical role in maintaining the health and functioning of an ecosystem; Berg et al., 2020; Fan et al., 2021) in agricultural systems can play a central role in community stability and improve soil fertility, nutrient cycling, and disease suppression (Li et al., 2017; Van Der Heijden & Hartmann, 2016). Introduction of microbial inoculants in some cases may encourage the growth and proliferation of keystone taxa, resulting in a more robust and resilient soil ecosystem (Agoussar & Yergeau, 2021; Li et al., 2020). Further, commercial exploitation of microbial inoculants requires an understanding of inoculant interactions with native soil microbial communities for potential ecological impacts and safety. Currently, we have a limited understanding of the mechanisms that underpin the impacts of microbial inoculants on plant growth and resilience to biotic and abiotic stresses. Understanding these mechanisms can provide the ability to identify inoculants with improved consistency and to modify either the composition of microbial inoculants or develop a better approach to application that can enhance their field performances.

As a principal global staple, the wheat crop forms a critical part of our food system. However, its production faces a potentially dramatic reduction of up to 30% due to shifting rainfall patterns triggered by climate change (Wang et al., 2020). Given the negative economic (e.g., cost of inputs) and environmental impacts of conventional farming practices, there is a pressing need to develop microbial‐based solutions for mitigating wheat drought stress. In this study, we performed a controlled mesocosm experiment to scrutinize the drought‐resistant performance of three microbial inoculants on three Australian wheat varieties using two contrasting soil types. Our study is underpinned by a robust sampling size, encompassing a total of 480 samples, combined with comprehensive analyses that integrated soil nutrients, microbial community structure, as well as plant phenotype data both before and after the implementation of drought treatments. By undertaking this experiment, we aimed to (i) identify the impact of microbial inoculants on the performance of different wheat varieties under drought conditions across two soil types, (ii) assess the impact of microbial inoculants on soil biological functions (e.g., enzymatic activities), biodiversity, and (iii) evaluate the colonization potential of microbial inoculants in soil by detecting their interactive relationships with indigenous soil microorganisms. Our findings enhanced our understanding of the interplay between soil, microbes, and crops under stress, providing compelling evidence for the potential use of bio‐inoculants in bolstering agricultural productivity under future climate scenarios.

## EXPERIMENTAL PROCEDURES

### Experimental design and set up

**FIGURE 1:**
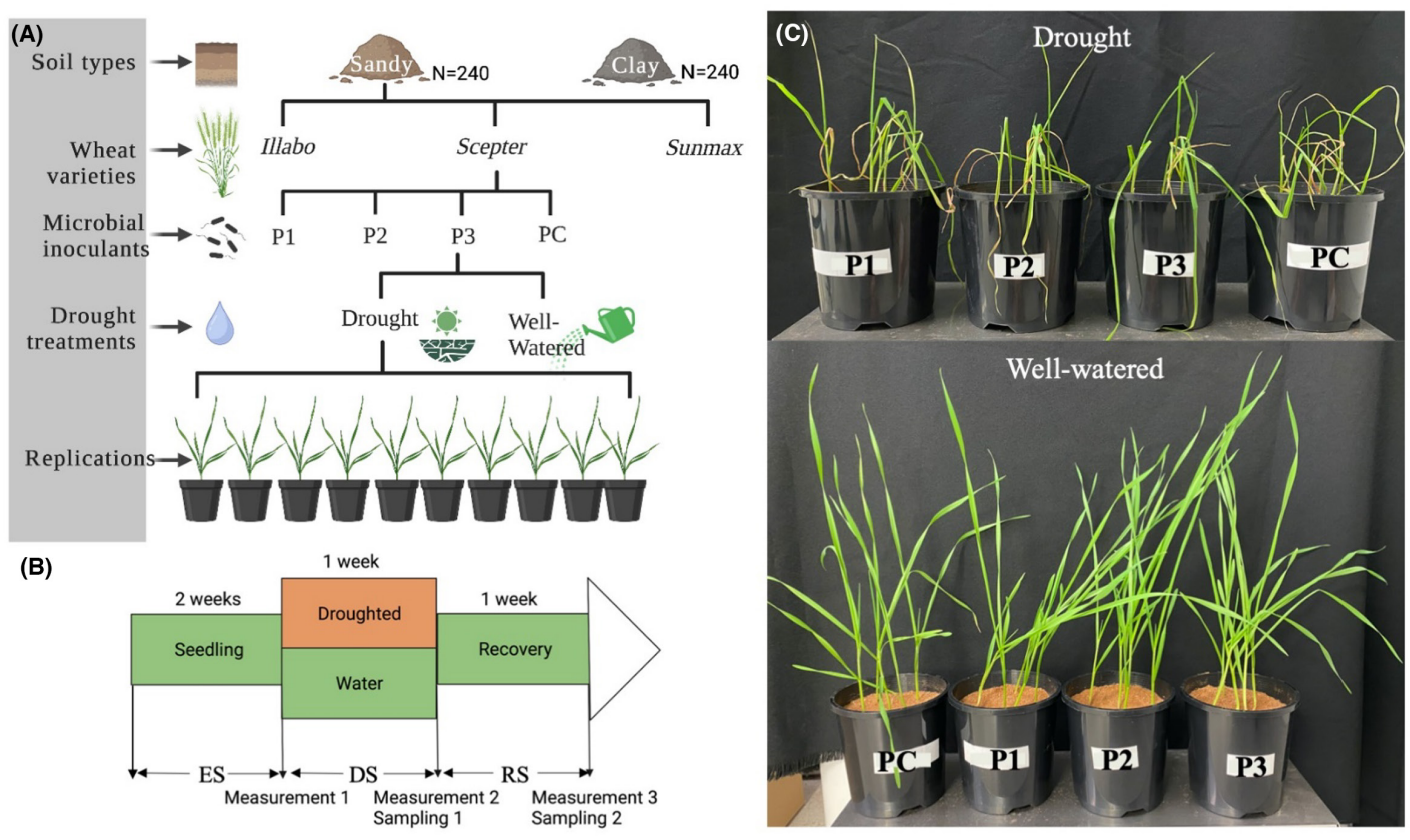
Illustration showing the experimental design. (A) A graphical representation of the experimental design that examines the effect of three distinct microbial inoculants on wheat growth under both well‐watered and drought conditions. The experiment employed two different soil types– sandy and clay – and tested three wheat varieties: *Illabo*, *Sceptre*, and *Sunmax*. (B) The parameters for plant growth were established at three stages: the establishment stage (ES), the drought stage (DS), and the recovery stage (RS). Destructive sampling took place following the DS and RS. In total, 480 samples were collected for soil analyses. All experimental activities were conducted under controlled conditions, maintaining a consistent temperature of 22°C and observing a 12:12 light/dark cycle. (C) Following the DS, we monitored and evaluated the wheat performance, focusing on understanding the drought impacts and the effectiveness of the microbial inoculants in promoting wheat growth.

The experiment was set in a controlled‐environment chamber at the Hawkesbury campus, Western Sydney University, Australia from December 2020 to January 2021. The experiment was a randomized complete block design using three wheat varieties (Sceptre, Illabo, and Sunmax), three microbial inoculant treatments (two bacterial consortia, P1 and P2 (Chr. Hansen, Denmark), one fungal inoculant, *Penicillium* sp. (P3) from wheat rhizosphere isolation and no microbial inoculant control (PC)), two drought treatments (drought and well‐watered), and two soil types (sandy and clay soil collected from Young, NSW; Figure 1). The properties of sandy soil were pH 5.72, total C 1.01%, total N 0.09%, and available P 0.62 mg/L and the properties of clay soil were pH 5.44, total C 1.87%, total N 0.17%, and available P 0.27 mg/L. The wheat varieties used in this study were Sceptre, Sunmax, and Illabo, which were the most widely used varieties in Western Australia and New South Wales, Australia. The experiment consisted of 10 replications for each treatment, as outlined in Figure 1A.

The two soils were sieved through a 2 mm mesh and then placed into small pots with a diameter of 115 mm, with 500 g of soil per pot. Each pot received mineral fertilizers at the rates of 60 kg N/ha, 40 kg K/ha, and 35 kg P/ha to ensure enough nutrients for wheat growth within the timeframe of this experiment. Then, seeds were sown into the soil, with two seeds per pot.

### Microbial inoculants treatments

We used two distinct microbial inoculant products, referred to as P1 and P2 in this study. Each of these products is a bacterial consortium comprising two distinct strains of *Bacillus* species, namely *Bacillus paralicheniforms* and *Bacillus subtilis*. Microbial inoculant P1 consists of *Bacillus paralicheniforms* (strain A) and *Bacillus subtilis* (strain X), while microbial inoculant P2 comprises *Bacillus paralicheniforms* (strain B) and *Bacillus subtilis* (strain Y). these microbes are part of nature and have been isolated from soils. The microbial inoculant products employed in this study were initially obtained in dry powder form. Prior to their application, these powdered products underwent a reconstitution process to create bacterial suspensions. This reconstitution was accomplished by diluting the dry powder with water at a ratio of 1:125 (weight to water), resulting in the formation of bacterial suspensions. The fungal inoculant P3 was sourced from the existing lab cultures, having been previously isolated from the wheat rhizosphere. The P3 cultures were cultured in potato dextrose broth (PDB) and prepared into a spore suspension with an optical density (OD) of 0.25 at 600 nm for inoculating wheat seeds. Prior to sowing, the seeds were coated by soaking them in a 100 mL suspension of microbial inoculants for 24 h.

### Drought treatments

Soil water status was estimated prior to the water application to pots by using the water holding capacity (WHC) as a metric. The drought treatments were applied using two distinct water levels: 80% WHC simulating well‐watered conditions and 30% WHC inducing drought conditions. Throughout an initial establishment stage (ES) lasting for 2 weeks, all pots were meticulously watered daily with tap water, maintaining 80% WHC. Following this stage, seedlings embarked on a drought stress treatment regime. During this drought (DS) stage, which spanned 7 days, the water level was kept at 30 ± 5% WHC in drought treatment pots, after which plants exhibited signs of severe wilting and leaf browning. Control pots were kept well‐watered, maintaining at 80 ± 5% WHC during the drought stage. Once the DS concluded, a recovery stage (RS) commenced, lasting for 7 days. During this phase, all pots were re‐watered to 80 ± 5% WHC, providing the plants and soil organisms a timeframe to recover. The experiment was marked by two sampling events: T1 = immediately following the drought stage, and T2 = upon conclusion of the recovery stage, as outlined in Figure 1B.

### Sampling and plant growth parameters measurements

Upon the completion of DS and RS, multiple components of plants and soils, including shoots, roots, bulk, and rhizosphere soil, were carefully collected with five replications, respectively. Bulk and rhizosphere soil was stored at −20°C for further analysis. To assess the impact of inoculant treatments and drought stress on wheat growth, shoot length, shoot diameter, and aboveground biomass (shoot weight) were measured via destructive sampling. Also, we evaluated the chlorophyll content of fully expanded leaves using a SPAD meter (Spectrum Technologies, Inc.). Lastly, the leaf temperature, an indicator of the plant's metabolic activity and response to environmental stress, was measured using an infrared thermometer (Agri‐Therm III Model 6110L; Everest Interscience, Inc.) from a distance of approximately 30 cm away from the leaf surface.

### Soil physiochemical and biological properties measurements

Soil samples at the end of DS were used for an extracellular enzyme activity assay. Soil moisture content was calculated by comparing the weight of a 10–20 g soil sample before and after oven drying at 105°C until it reached a constant weight. To determine the soil total carbon and total nitrogen content, 0.5 g of 70°C oven‐dried and milled soil was analysed using a LECO TruMac CN analyser (LECO cooperation). The soil was air dried at 40°C for the available phosphorus (AP) assay. Then 1.0 g of soil was extracted in 100 mL of sodium bicarbonate, shook at 800 rpm for 16 h, filtered by Whatman filter paper, acidified with 2 mL of 12 N sulphuric acid to a 40 mL portion of soil extract, and measured on an AQ2 discrete analyser (Jayaramaiah et al., 2022). Soil pH was measured by combining soil and water in a 1:2.5 ratio, shaking for 1 h at 180 rpm, centrifuging 2 min at 2000 rpm, and using a Delta pH meter (Mettler‐Toledo Instruments). Finally, extracellular enzyme activities associated with total carbon, nitrogen, and phosphorus were measured according to the method described by Bell et al. (2013). We quantified the potential activities of six soil hydrolytic enzymes using fluorometric techniques. These enzymes encompassed four involved in labile‐C cycling (α‐1,4‐glucosidase, β‐1,4‐glucosidase, β‐1,4‐xylosidase, and cellobiohydrolase), one related to nitrogen cycling (β‐1,4‐N‐acetyl‐glucosaminidase), and one associated with phosphorus cycling (acid phosphatase). Briefly, we initiated the process by homogenizing 2.75 g of soil in 45 mL of 50 mM sodium acetate buffer (pH 5.5) using a Waring blender, for a duration of 1 h. Following homogenization, we transferred 200 μL of soil slurry into individual wells of a 96‐well microplate. Each well was then supplemented with 50 μL of a 200 μM fluorometric substrate, a concentration chosen to saturate the reaction. Subsequently, the microplates were incubated in darkness at a constant temperature of 25°C for a period of 6 h. After incubation, we quantified the fluorescence using a fluorescence spectrometer (Spectramax M2; Molecular Devices) with excitation and emission wavelengths set at 365 and 450 nm, respectively. Enzyme activity was expressed as nanomoles per gram of dry weight per hour (nmol activity/g dry soil/h).

### Soil microbial community characterization

Soil samples from DS were profiled by DNA sequencing to characterize microbial communities. Metagenomic DNA was extracted from 0.25 g of soil using the Qiagen DNeasy PowerSoil Pro DNA extraction kit (QiagenInc.) according to the manufacturer's instructions. Primer pairs 799f/1193r and fITS7/ITS4 targeting the bacterial 16S rRNA gene and the fungal internal transcribed spacer (ITS region) were used in amplicon sequencing to characterize bacteria and fungi communities, respectively (Naylor et al., 2017). High‐throughput sequencing was performed on an Illumina MiSeq platform (2* 300 PE) at the Next Generation Sequencing (NGS) Facility of Western Sydney University. Bioinformatic analysis on the raw sequencing data from NGS was performed using pipelines combining USEARCH (Edgar, 2010) and QIIME2 (Bolyen et al., 2019). Briefly, USEARCH was used to remove low‐quality reads, merge pair‐ended reads, and dereplication. Operational taxonomic Unit (100% sequencing identity OTU) clustering and chimeric filtering were performed using UNOISE3 (Edgar & Flyvbjerg, 2015). The resultant representative sequences of OTU were then aligned against the SILVA v138 database (Quast et al., 2012) and the UNITE database (Nilsson et al., 2018) in QIIME2 for bacteria and fungi taxonomic information, respectively. Resultant OTU tables were normalized at 8000 reads for bacteria and 6000 reads for fungi per sample before downstream analysis.

### Statistical analysis

To assess the effects of microbial inoculants on wheat growth and soil biological functions, we conducted one‐way analyses of variance (ANOVA) on various parameters, including shoot and root weight, shoot length, leaf temperature, and chlorophyll content. Microbial inoculant treatments were used as the primary factor in the ANOVA. We also calculated significant differences in soil microbial communities across different soil types, wheat varieties, and microbial inoculant treatments using Permutational multivariate analysis of variance (PERMANOVA). All statistical analyses were performed using R Statistical Software 3.5.1 (R Core Team, 2022). A *p*‐value of 0.05 or less was considered statistically significant, and means were considered significantly different at this level. Differential OTU analysis was performed using the R package DEseq2 (Love et al., 2014) to find out significantly different OTU among microbial inoculant treatments.

Network analysis was performed using the Cytoscape plugin CoNet (Faust et al., 2012). Prior to calculation, OTUs with less than four occurrences and 10 reads were filtered out to minimize spurious correlations of rare taxa. Using 15 replicates, five different correlation algorithms including Spearman correlation, Pearson correlation, Bray‐Curtis distance, Kullback‐Leibler distance and Mutual Information were calculated, and correlations with only one algorithm supported were excluded (Hu et al., 2016). The Brown *p*‐value combination algorithm was used for *p*‐value merging. The Benjamini‐Hochberg (BH) multiple tests with 100 bootstraps and permutations were used to control the false positive rate (FPR, *p* < 0.001; Bazany et al., 2022). Topological properties of the resultant network, including node degree of connectivity, closeness, and betweenness centrality, were calculated in Gephi 9.0 (Bastian et al., 2009).

## RESULTS

### Wheat growth parameters

Wheat growth parameters were recorded at three distinct stages. Prior to drought treatment (on ES), in sandy soil, microbial inoculant P1 increased shoot length significantly by 12.9% (*p* < 0.0001), but no significant effect of P1 on wheat shoot length was detected in clay soil (Table S1).

**FIGURE 2:**
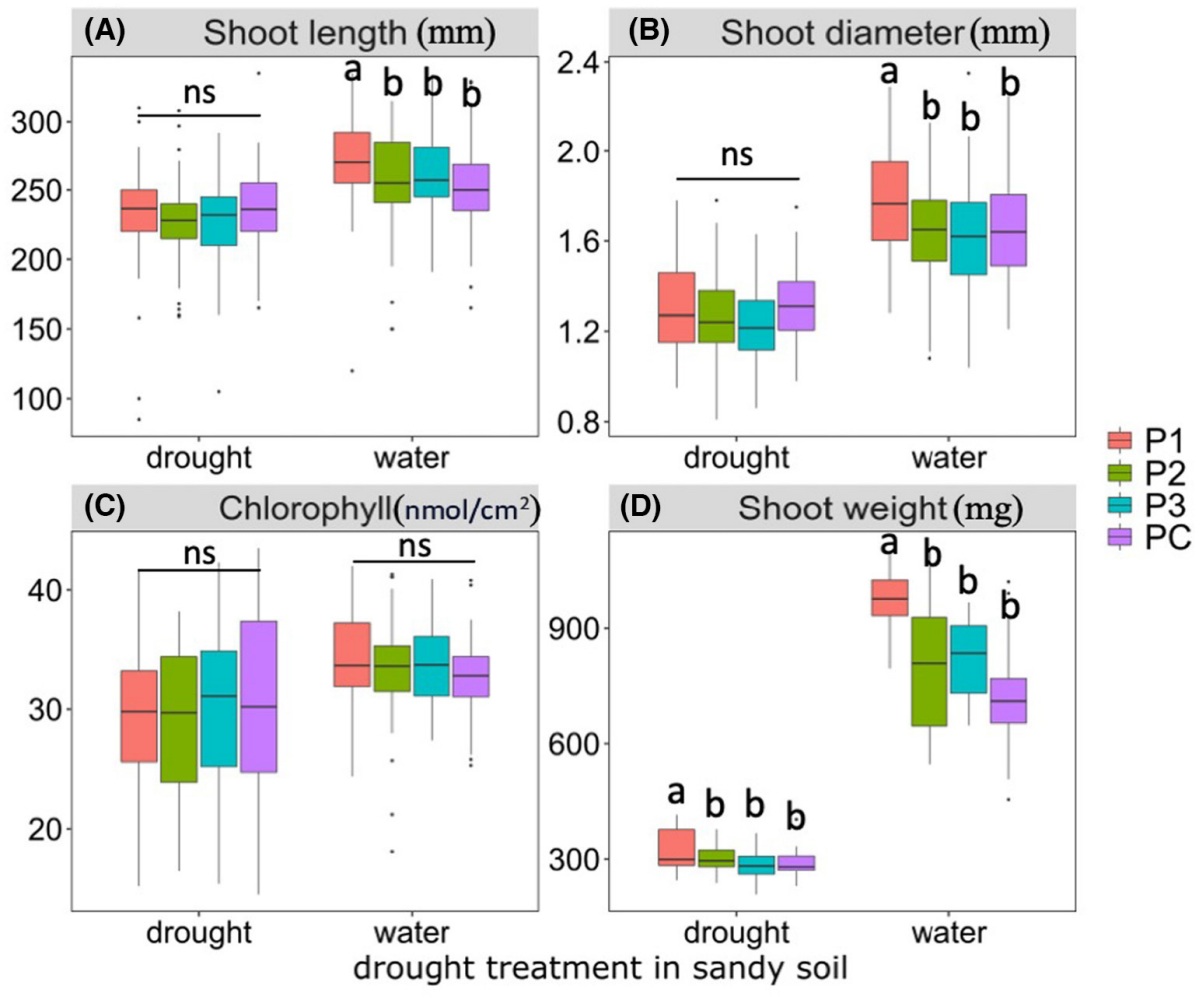
Effects of microbial inoculant treatments on wheat phenotype after drought stress. Figures represent (A) shoot length, (B) shoot diameter, (C) chlorophyll content, and (D) shoot weight of wheat (including all varieties) under both drought and well‐watered conditions in sandy soil. In these graphs, distinct letters represent groups with significant differences, while ‘ns’ denotes no significant difference among groups. The middle back lines inserted in bars represent median values for each treatment, with the upper and lower lines representing the first (25th percentile) and third (75% quantile) quantiles of the data points. *N* = 5 for each treatment.

At the end of the drought stage (DS), the adverse impact of drought treatment on wheat growth was evident in both soil types (*p* < 0.01), as shown by the decreased shoot length, shoot diameter, and weight (Figure 2). In sandy soil, under well‐watered condition, microbial inoculant P1 significantly promoted wheat shoot length (by 6.7%, *p* = 0.006) (Figure 2A), diameter (by 8.5%, *p* = 0.002) (Figure 2B), and weight (by 33%, *p* = 0.000; Figure 2D) compared with PC (the control). Under drought condition, shoot length, and diameter were unaffected by microbial inoculant treatments, but shoot weight was significantly increased (11.37%, *p* = 0.024) by the P1 treatment compared with PC (Figure 2D) in sandy soil. No significant differences were observed in chlorophyll level and leaf temperature between the microbial inoculants and control under drought condition in sandy soil (Tables S1 and S2). In clay soil, the microbial inoculants had less influence on wheat growth than had been observed in sandy soil, regardless of whether the conditions were drought‐ or well‐watered. In well‐watered clay soils, P1 significantly increased shoot length (6.86%, *p* = 0.036; Figure S1a) across three varieties and chlorophyll (9.45%, *p* = 0.000) of Sceptre (Table S2), while in drought clay soils, plant growth parameters were unaffected by the inoculant treatment (Figure S1). Among the three wheat varieties tested in sandy soil, Sunmax was the most responsive to P1 inoculant (Figure S2, Table S2), whose shoot weight (36.33%, *p* = 0.009) was significantly increased under drought treatment. By contrast, Illabo and Sceptre were less responsive to the P1 inoculant than Sunmax. However, no significant effects were found on the three varieties across the inoculant treatments in clay soil (Figure S3).

After RS, in sandy soil, both P1 and P2 increased shoot weight significantly under well‐watered conditions (*p* = 0.003; *p* = 0.024). No significant effects were observed in shoot weight across the microbial inoculants for drought treatment in sandy soil. Similarly, there was no significant difference on wheat growth across the inoculant treatments in clay soil (Table S1).

### Soil extracellular enzyme activities

**FIGURE 3:**
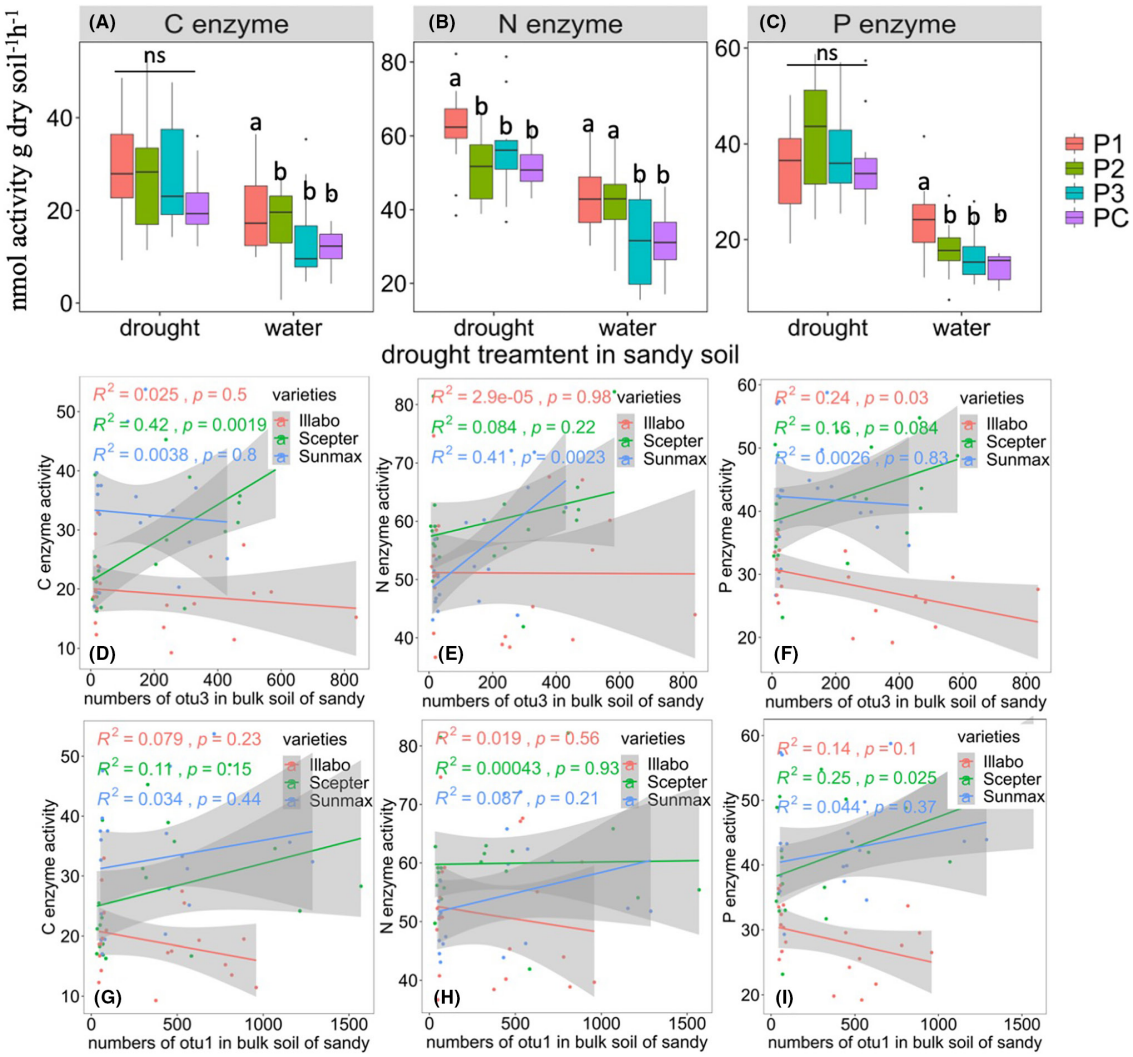
Effects of microbial inoculants on potential soil extracellular enzyme activities after drought stress. The graphs depict changes in (A) Carbon (C) enzyme activity, (B) Nitrogen (N) enzyme activity, and (C) Phosphorus (P) enzyme activity under both drought and well‐watered conditions in sandy soil. The correlation between the number of inoculant OTUs and soil enzyme activities in sandy bulk soil. This includes (D) the number of otu3 correlated with C enzyme activity, (E) the number of otu3 correlated with N enzyme activity, (F) the number of otu1 correlated with P enzyme activity, (G) the number of otu1 correlated with C enzyme activity, (H) the number of otu3 correlated with N enzyme activity, and (I) the number of otu1 correlated with P enzyme activity. In the graphical representation, different letters indicate significance between different groups, while ‘ns’ denotes no significant differences among the groups. The middle back lines inserted in the bars represent the median values for each treatment. Furthermore, the upper and lower lines depict the first (25th percentile) and third (75% quantile) quartiles of the data points, respectively. *N* = 5 for each treatment.

Extracellular soil enzyme activities were assayed after the DS stage. In sandy soil, under well‐watered condition, microbial inoculants especially, P1, significantly increased total C enzyme activity (58.3%), total N enzyme activity (32.3%), and total P enzyme activity (53.3%), while P2 increased total N enzyme activity by 35.5% compared to the PC (Figure 3). Under drought conditions, inoculation with P1 increased total N enzyme activity by 21.6% compared to the control. However, in clay soil, no significant differences were observed in the microbial inoculant treatments under drought or well‐watered conditions (Figure S4). When wheat varieties were considered separately under drought condition the addition of P1 significantly enhanced total C enzyme activity (116.7%) in soil with Sceptre and total N enzyme activity in soil with Sunmax compared to the control (Table S3) in sandy soil. P2 treatment showed a significant increase in total C enzyme activity (73.9%) in soil with Sunmax compared to the control in sandy soil.

Two OTUs identified as OTU1 and OTU3 were found to correspond to *B. paralicheniformis* and *B. subtilis* based on sequence homology and were identical to microbial inoculants P1 and P2. Regression analysis between the inoculant OTUs and soil enzyme activities showed a strong correlation between microbial inoculants on soil functions in sandy bulk soil. It revealed a significant increase in C enzyme activity of Sceptre (*R* ^2^ = 0.42, *p* = 0.0019) (Figure 3D) and N enzyme activity of Sunmax (*R* ^2^ = 0.41, *p* = 0.0023) (Figure 3E) associated with the abundance of OTU3. Furthermore, OTU1 demonstrated a significant positive effect on P enzyme activity of Sceptre (*R* ^2^ = 0.25, *p* = 0.0025; Figure 3I).

### Relationship between microbial inoculants, wheat phenotype, and soil microbial community

**FIGURE 4:**
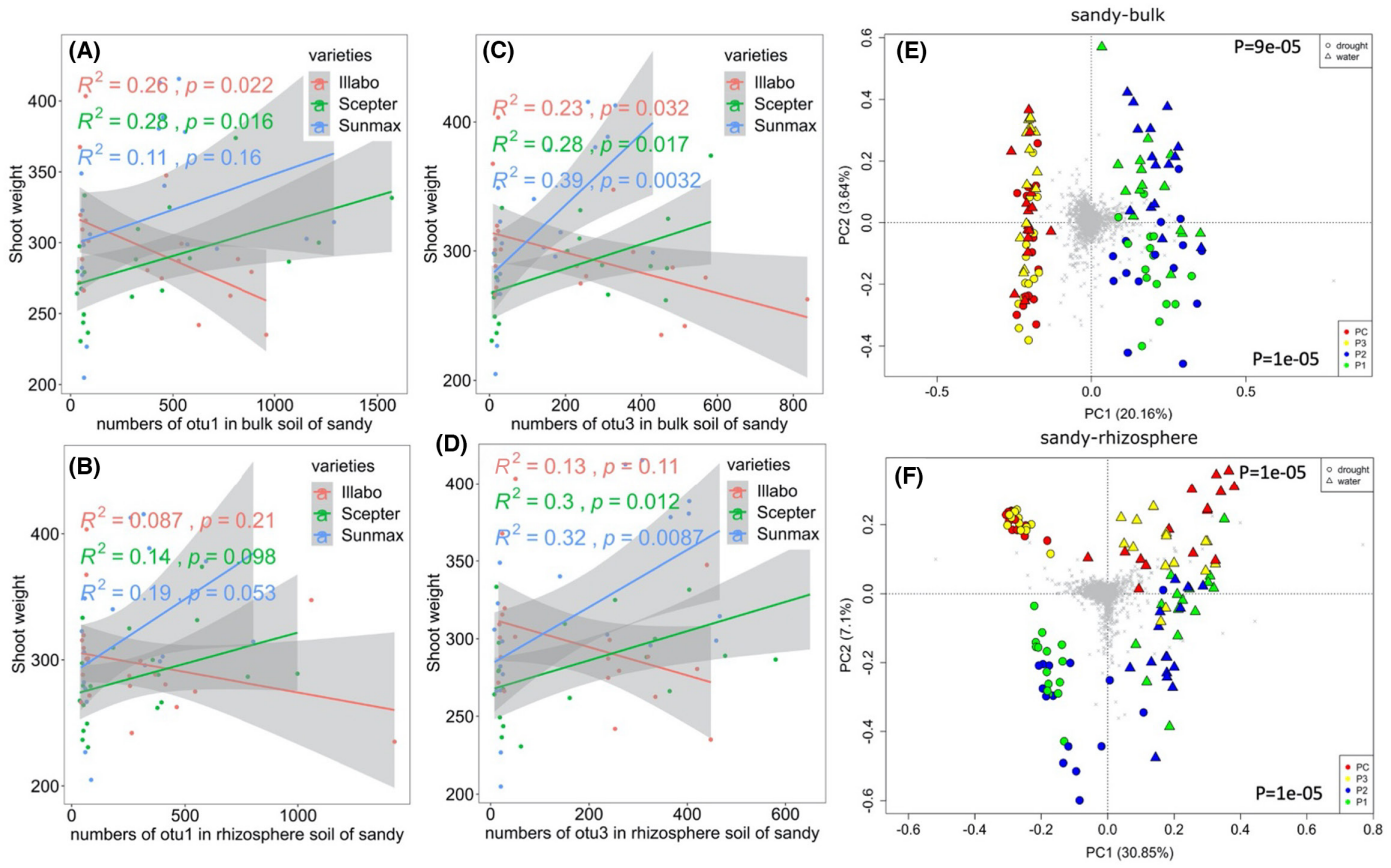
Principal analyses (PCA) summarizing the effects of microbial inoculants on the soil bacterial community and correlations between inoculant OTUs and shoot weight of three wheat varieties after drought stress. Displayed are regression line graphs illustrating the relationship between shoot weight of three wheat varieties and the abundance of specific operational taxonomic units (OTUs) in (A) sandy bulk soil (OTU1), (B) sandy rhizosphere soil (OTU1), (C) sandy bulk soil (OTU3), and (D) sandy rhizosphere soil (OTU3). Principal Component Analysis (PCA) plots illustrate the bacterial community composition in (E) sandy bulk soil, and (F) sandy rhizosphere soil. The *p*‐values shown on the top and bottom of the plots represent statistical significances observed in soil microbial communities between drought treatments and microbial inoculant treatments and a significant relationship between inoculant OTUs and shoot weight. Each grey cross within the plot represents a distinct Operational Taxonomic Unit (OTU) detected in the bacterial communities of corresponding soil compartments. *N* = 5 for each treatment.

A regression revealed that the abundance of OTU1 in the bulk soil exhibited a positive association with the shoot weight of Sceptre (*R* ^2^ = 0.28, *p* = 0.016; Figure 4A). However, the abundance of OTU1 in the rhizosphere soil did not show a significant association with shoot weight across the three wheat varieties (Figure 4B). Furthermore, the abundance of OTU3 in the bulk soil showed a positive association with the shoot weight of Sceptre (*R* ^2^ = 0.28, *p* = 0.017) and Sunmax (*R* ^2^ = 0.39, *p* = 0.0032) (Figure 4C). In the rhizosphere soil, the abundance of OTU3 was also positively associated with the shoot weight of Sceptre (*R* ^2^ = 0.3, *p* = 0.012) and Sunmax (*R* ^2^ = 0.32, *p* = 0.0087; Figure 4D).

Our results identified five factors that impacted the diversity and structure of soil bacterial and fungi communities (Tables S4 and S5), with soil types being the most influential, followed by soil compartments and drought treatments (*p* < 1.00E‐05). While microbial inoculants had a significant (*p* < 1.00E‐05) impact on the soil bacterial community, there was no significant impact observed on the soil fungal community. No significant difference was found among the different varieties.

In sandy soil, the results of PCA supported that drought and microbial inoculants are two significant factors that influence soil microbial communities. Microbial inoculants P1 and P2 had a greater impact than drought stress on shaping the microbial community structure in bulk soils (Figure 4A). Specifically, the inoculant treatments (P1 and P2) resulted in the separation of bacterial communities into two distinct groups along the first axis (P1 and P2 forming one group, while P3 and PC forming the other group; *p* < 1.00E‐05). Additionally, drought stress emerged as the primary factor dividing the bacterial community into two groups (drought treatment forming one group, while well‐watered treatment forming the other group) along the second axis (*p* < 9E‐05). However, in the rhizosphere soil, the bacterial communities showed a separation based on drought conditions along the first axis, while the microbial inoculants P1 and P2 accounted for the separation along the second axis (Figure 4B). The fungal communities in the P3 treatment were separated from the others along the first axis in bulk soil (Figure S5a).

In clay bulk soil, the bacterial communities exhibited a clear separation on the first axis, primarily driven by the presence of microbial inoculants P1 and P2, distinct from the other microbial inoculant treatments (Figure S6a). Additionally, the fungal communities did not show any separation based on microbial inoculants in either bulk or rhizosphere soil (Figure S7a,b).

### Microbial composition and indicator microbial species

**FIGURE 5:**
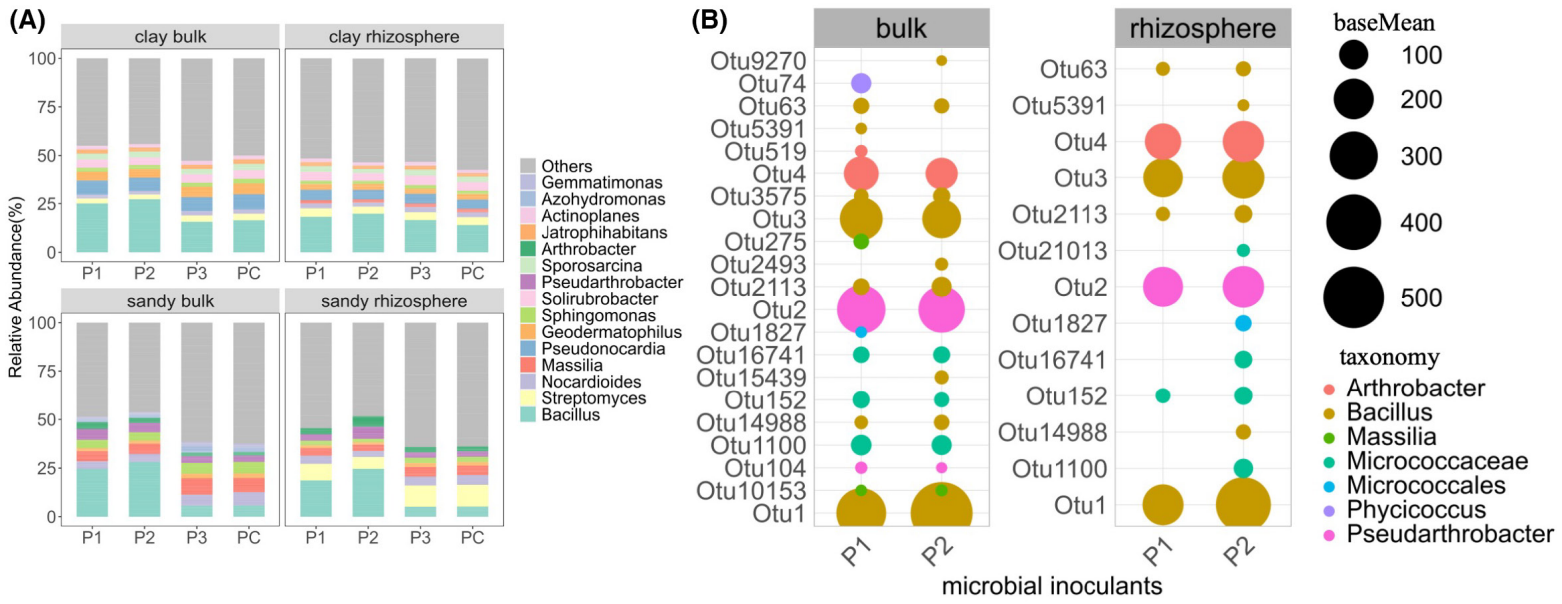
Impacts of drought stress on soil bacterial composition at genus level and biological markers under drought stress. Two key aspects are presented: (A) the community composition of soil bacteria at the genus level across different compartments, soil types, and microbial inoculant treatments; and (B) the significantly enriched Operational Taxonomic Units (OTUs) observed in P1 and P2 treatments compared with the PC treatment in both the bulk soil and rhizosphere of sandy soil under drought conditions.

Microbial composition and differential OTU analysis were conducted under drought condition after DS. It indicated that the microbial inoculants (P1 and P2) easily colonized the soil, as OTU1 and OTU3 (blasted to be *B. paralicheniformis* and *B. subtilis*) had very high abundance in bacterial communities. However, fungal inoculant P3 did not establish a significant presence in the soil, as it did not exhibit high abundance within the fungal communities. In sandy soil, *Bacillus* became the dominant bacterial genus under P1 and P2 treatment, while in the control, *Massilia* and *Streptomyces* were the most abundant in bulk and rhizosphere soil, respectively (Figure 5A). In clay soil (Figure 5A), *Bacillus* was the most abundant genus in all groups in both bulk and rhizosphere soil. As for fungi (Figure S8), *Fusarium* was dominant across the groups in the bulk soil of sandy and clay, and microbial inoculants had no significant impact on the composition of fungal community. While *Chaetomium* was the prevalent genus in the rhizosphere of sandy soil without a significant difference among the microbial‐inoculant treatments.

Additionally, in sandy soil, differential OTUs analysis was conducted on bacterial communities between the microbial inoculant treatments (P1 or P2) and PC treatments, and analysis on fungi communities between P3 and PC treatments. The number of OTUs increased significantly with P1 and P2 treatments compared with PC in the bulk and rhizosphere soils (Figure S9, Table S6). For example, in the P1 and P2 treatments, OTU2 (*Pseudarthrobacter*) and OTU4 (*Arthrobacter*) significantly increased (*p* < 0.05) compared to the PC in both bulk and rhizosphere soil (Figure 5B), while OTU9 (*Massilia*) and OTU17 (*Sphingomonas*) significantly decreased (*p* < 0.05) in bulk soil (Figure S9a,b) compared to the PC treatment. The P3 treatment did not have a significant impact on the fungal composition (Figure S9e,f). We only found one OTU, OTU159 (*Aspergillus nidulans*), that increased significantly (*p* = 8.84E‐06) in the P3 treatment compared to the PC treatment in both bulk and rhizosphere soil. Furthermore, P1 and P2 induced many new species in Bacillus compared to PC, such as *Bacillus nakamurai*, *Bacillus aestuarii*, *Bacillus solani*, and *Bacillus siamensis* (Table S7).

### Microbial co‐occurrence network

The network analysis revealed distinct network properties across various compartments and microbial inoculant treatments (Figure S10, *n* = 15) in sandy soil under drought condition. Adding microbial inoculants led to the development of more complex network relationships in both bulk and rhizosphere soil, as determined by the topological properties including nodes, edges, average degree, etc. The most complex interactions were observed in the P2 treatment.

**FIGURE 6:**
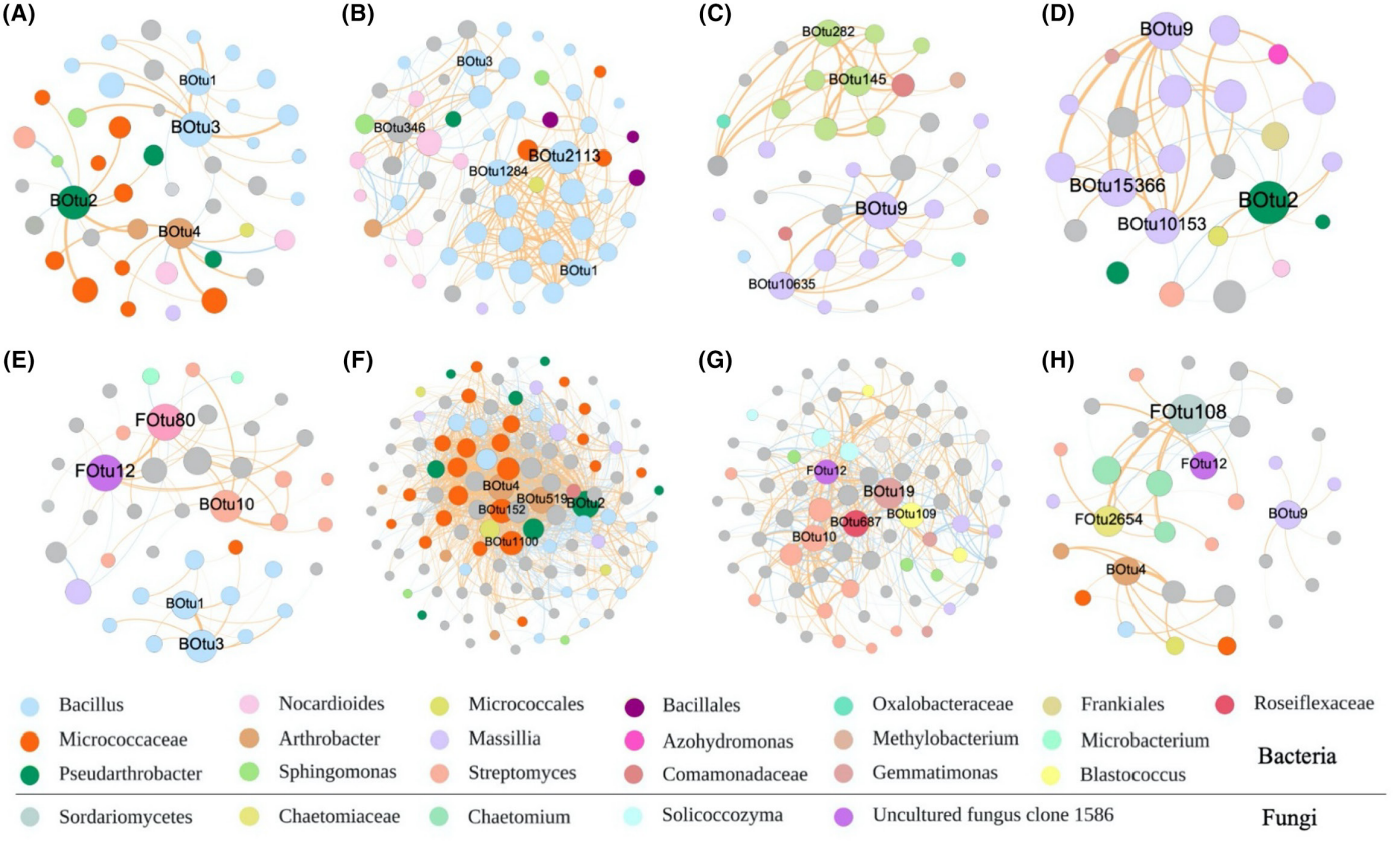
Microbial co‐occurrence network analyses summarizing potential roles of bio‐inoculants in shaping soil microbial structure and the implications for soil functions. This analysis was carried out after the drought stage (DS), examining the network of hub zOTUs with the top four degrees in sandy soil under drought conditions. The network structures for each microbial inoculant treatment in bulk soil and rhizosphere soil are separately displayed: (A) P1 treatment in bulk soil; (B) P2 treatment in bulk soil; (C) P3 treatment in bulk soil; (D) PC treatment in bulk soil; (E) P1 treatment in the rhizosphere soil; (F) P2 treatment in the rhizosphere soil; (G) P3 treatment in the rhizosphere soil; and (H) PC treatment in the rhizosphere soil. In these networks, circles of the same colour represent the same taxa. BOtu refers to bacteria, and FOtu refers to fungi.

Keystone taxa (the top four OTUs with the highest degree in each network) and OTUs that were directly linked with them were further extracted from the complete network (Figure 6). In the P1 treatment, OTU1 and OTU3 (P1) emerged as the most influential keystone taxa in the bulk (Figure 6A) and rhizosphere (Figure 6E) of sandy soil, followed by OTU2 and OTU4 in the bulk soil. However, in P2 treatment, the most influential keystone microbe was OTU2113 (*Bacillus*), followed by OTU1 and OTU1284 (*Bacillus*) in the bulk soil (Figure 6B). The most influential keystone taxa in the rhizosphere soil were OTU519 (*Arthrobacter*) and OTU2 (Figure 6F). In the P3 treatment, OTU9 was the most influential keystone microbe in the bulk soil (Figure 6C), and OTU9 and OTU2 were the most influential keystone taxa in the control group in the bulk soil (Figure 6D).

To trace the microbes that interacted with the microbial inoculants, we highlighted all the OTUs that had a positive or negative relationship with OTU1 and OTU3 (Figure S11). Our analysis revealed that in both the P1 and P2 treatments, OTU1 and OTU3 had a very strong positive connection with Bacillus in both bulk and rhizosphere soil. Additionally, some members of the Micrococcaceae family had a positive relationship with them in both bulk and rhizosphere soil, while Streptomyces had a negative relationship with them in the rhizosphere soil.

## DISCUSSION

Drought is a major source of stress for crops such as wheat, which rely on adequate water availability for optimal growth and yield production. Microbial inoculants are increasingly considered a viable and promising approach to counteract the effects of drought on crops. Our study revealed that both P1 and P2 were able to colonize bulk and rhizosphere soils, while P3 was not. This highlights the fundamental steps of successful colonization for impactful inoculation: P3 lacked effective colonization, which was reflected in its non‐impact on soil or plant performance. Additionally, our study found differences in functional strains can affect the efficiency of inoculants. P2 was able to colonize but did not provide the same level of host functions to mitigate drought as P1. We observed that P1 (*Bacillus*) significantly increased plant weight under drought conditions. This finding is supported by previous research, which reported improved growth and water‐use efficiency in wheat plants inoculated with *Bacillus subtilis* under drought conditions (Gagné‐Bourque et al., 2016). Another study revealed that wheat plants inoculated with *Bacillus paralicheniformis* and grown under water‐limited conditions produced greater biomass and higher gain yields compared to non‐inoculated plants (Valenzuela‐Ruiz et al., 2019). Our study advanced this discipline by providing evidence that the effects of inoculants are influenced by soil types and, to a lesser extent, crop cultivars. *Bacillus* is a versatile genus of microorganisms capable of thriving in natural soil environments due to its adaptability to a range of conditions. *Bacillus* spp. possess unique structural and metabolic traits that enable them to survive, including the ability to produce a multilayered cell wall structure, antibiotic secretion, and the production of stress‐resistant endospores. They can work in several ways to help plants resist drought stress. For example, they can produce enzymes that aid in plant nutrient acquisition and utilization (Ibarra‐Villarreal et al., 2021; Kaushal & Wani, 2016). Some researchers showed that *Bacillus subtilis* Rhizo SF48, which produced ACC deaminase, was able to protect tomato plants from oxidative damage caused by drought stress and promote plant growth (Gowtham et al., 2020). Additionally, some inoculants contribute to the efficient breakdown of organic matter, nutrient cycling, and the release of essential elements for plant growth, thereby enhancing soil structure, nutrient availability, and overall soil health. Moreover, it fosters enhanced water retention capacity, facilitating improved water uptake and utilization by plants (Costa et al., 2018; Gupta et al., 2015). Our results provide empirical support for such mechanisms, showing an increase in enzyme activity associated with N‐cycling in soils inoculated with P1. Simultaneously, wheat biomass under water‐limited condition was higher, implying that P1 contributed to increased drought resistance in the wheat plants. Other inoculants that boosted soil enzyme activity can further help plants access nutrients, thereby improving resilience under drought conditions (Alori et al., 2017; Owen et al., 2015).

During drought conditions, soil moisture levels decrease, potentially curtailing microbial activity and altering the composition of microbial communities (Bastida et al., 2017; Ochoa‐Hueso et al., 2018). In combination, the effects of microbial inoculants and drought on soil microbial communities can respond to a range of factors, such as the type of inoculants, the severity of the drought, as well as the soil and crop types (Breitkreuz et al., 2021; Qi et al., 2022; Wang et al., 2018; Yaghoubi Khanghahi et al., 2022). Previous reports suggested that microbial inoculants can significantly alter bulk and rhizosphere communities through processes of synergism or antagonism with indigenous microbes in the soil (Mawarda et al., 2020; Wang, Chen, et al., 2021; Wang, Li, et al., 2021). One of the potential benefits of microbial inoculants is their ability to transform into keystone taxa in the soil after inoculation, which can have a significant impact on microbial functions. In this study, inoculant P1 was established as the keystone taxa (OTU1 and OTU3) in both the bulk and rhizosphere of sandy soil, resulting in improved wheat performance under drought conditions. This provides preliminary evidence for effective colonization, establishment, and functional support from this inoculant, which resulted in drought tolerance in the crop. This also provides support for the argument that, for the consistent effect of microbial inoculants, every product should be evaluated through a metacommunity ecological framework (Singh et al., 2023). A reasonable explanation of the insignificant impact of P2 and P3 on wheat growth could be attributed to the lack of strong colonization and establishment (e.g., becoming a keystone taxon) in soil microbial communities.

Research has indicated that microbial inoculants can foster a positive relationship with other microbes in the soil, particularly those of the same genus, a concept supported by recent research (Finkel et al., 2017). When microbial inoculants are added to the soil, they can interact with the existing microbial communities in a variety of ways. In some cases, the microbial inoculants may compete with the native microbes for resources, which can result in a decrease in the abundance of these native microbes (Qiu et al., 2019). However, in other cases, the microbial inoculants may interact with the same genus of microbes in a positive way, which can enhance the overall functioning of the soil ecosystem (Liu et al., 2022). This positive relationship between microbial inoculants and the same genus of microbes in the soil highlights the potential of microbial inoculants to work in concert with existing microbial communities, resulting in a more robust and resilient soil ecosystem. Microbial inoculants could simultaneously interact with existing microbial communities in the soil and promote the growth of rare species in some cases (Shi et al., 2022). Our results suggest that P1 may potentially increase in the relative abundance of other strains of *Bacillus*. Different strains of the *Bacillus* may interact to influence ecosystem and host functions, but empirical evidence for such interactions is needed (Alonso et al., 2020; Sanchez‐Gorostiaga et al., 2019). However, it is important to highlight that our work was carried out in environmentally controlled glasshouse, and previous results suggest a differential response of the same treatment between controlled environments and filed conditions (Li et al., 2022; Qiu et al., 2019). In a field condition with fluctuating climatic soil conditions and agronomic practices can influence the performance of microbial inoculants. Therefore, field trials are needed to confirm our findings before wide‐spread use of these microbial products.

Overall, our results indicate that P1 has the potential to effectively enhance wheat growth in sandy soil, especially under conditions of low moisture availability. Furthermore, it increased enzyme activity in the soil under drought conditions. This inoculant was a strong colonizer of the bulk and rhizosphere soils but was influenced by soil types and crop cultivars. Additionally, soil microbial communities were influenced by many factors, including soil types, drought, microbial inoculants, and soil compartments. Our study provides empirical evidence that effective colonization and establishment by microbial inoculants is the first important step to provide benefits for crops, supporting the argument that an ecological framework (e.g., metacommunity theory) should be incorporated into evaluating and predicting the efficiency of microbial inoculants. In conclusion, microbial inoculants showed promise as a tool for helping wheat plants resist drought stress, and if such performance is replicated in field conditions, such microbial inoculants could be a valuable addition to farmers' toolkits for managing drought risk with agriculture productivity.

## AUTHOR CONTRIBUTIONS

**Brajesh K. Singh:** Conceptualization (lead); data curation (equal); formal analysis (equal); funding acquisition (lead); investigation (equal); methodology (equal); project administration (lead); resources (lead); supervision (lead); validation (supporting); visualization (supporting); writing – review and editing (equal). **Hongwei Liu:** Data curation (supporting); formal analysis (supporting); investigation (supporting); validation (supporting); writing – review and editing (equal). **Jiayu Li:** Conceptualization (equal); data curation (lead); formal analysis (lead); investigation (lead); methodology (equal); validation (lead); visualization (lead); writing – original draft (lead); writing – review and editing (equal). **Catriona A. Macdonald:** Conceptualization (equal); investigation (supporting); supervision (supporting); writing – review and editing (equal). **Juntao Wang:** Conceptualization (lead); data curation (supporting); investigation (supporting); writing – review and editing (equal).

## FUNDING INFORMATION

No funding information provided.

## CONFLICT OF INTEREST STATEMENT

None declared.

## Supporting information


Figure S1

Table S1

Table S2

Table S3

Table S4

Table S6

Table S7
