# Supplementary material for: Microbial inoculants with higher capacity to colonize soils improved wheat drought tolerance
Source: Microb Biotechnol. 2023 Oct 10;16(11):2131–44. doi: 10.1111/1751-7915.14350 (PMC10616649; doi:10.1111/1751-7915.14350)
Supplement: Supplementary file 1 — Figure S1 [file MBT2-16-2131-s002.docx]

**Figure S1 Effects of microbial inoculant treatments on wheat phenotype after drought stress.** Figures represent (a) shoot length, (b) shoot diameter, (c) chlorophyll content, and (d) shoot weight of wheat (including all varieties), under both drought and well-watered conditions in clay soil. In these graphs, distinct letters represent groups with significant differences, while ‘ns’ denotes no significant difference among groups. Middle back lines inserted in bars represent median values for each treatment, with the upper and lower lines representing the first (25^th^ percentile) and third (75% quantile) quantile of the data points. N=5 for each treatment.

**
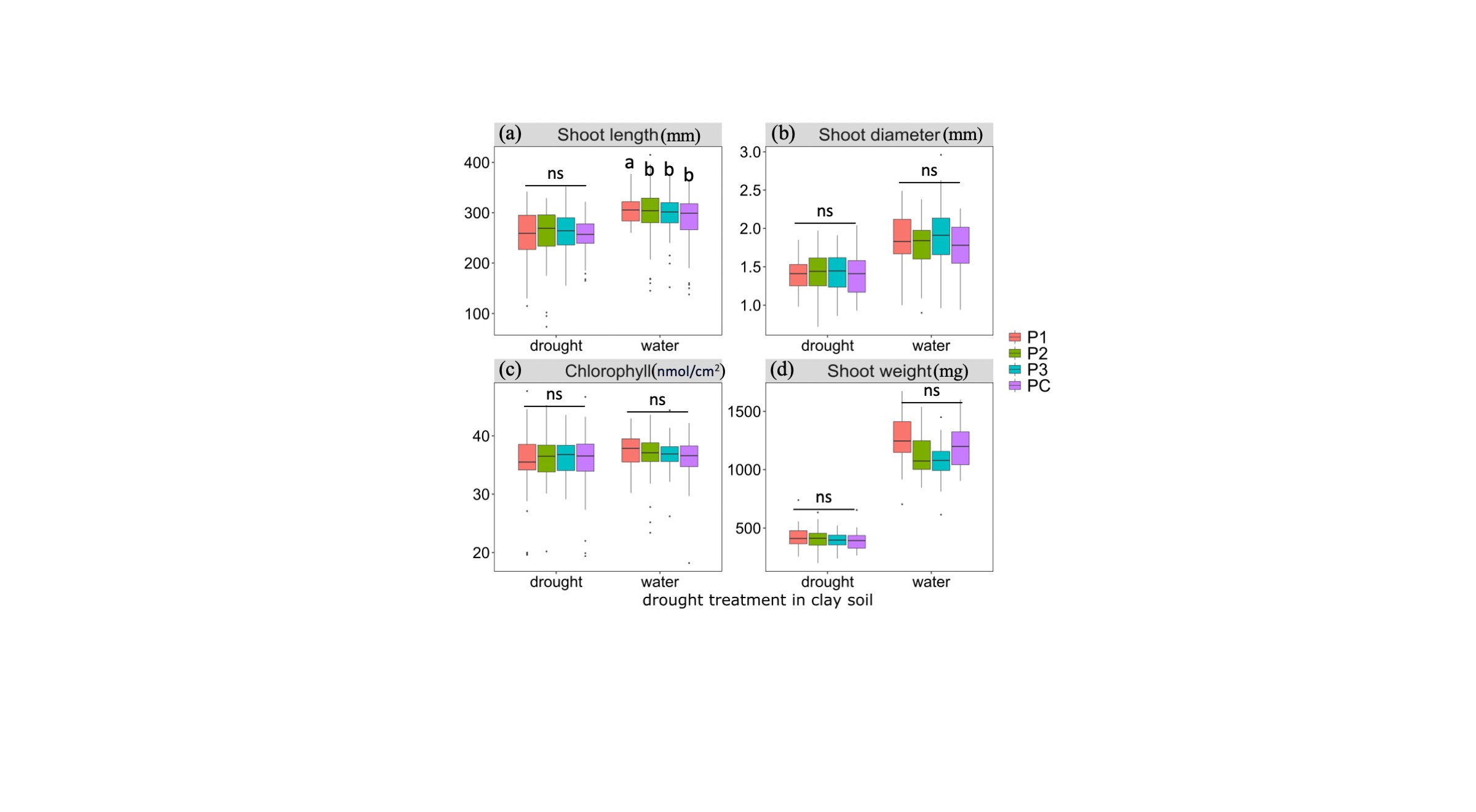
**

**Figure S2 Effects of microbial inoculant treatments on wheat phenotype after drought stress.** Figures represent (a) shoot length, (b) shoot diameter, (c) chlorophyll content, and (d) shoot weight of three wheat varieties under both drought and well-watered conditions in sandy soil. In these graphs, distinct letters represent groups with significant differences, while ‘ns’ denotes no significant difference among groups. Middle back lines inserted in bars represent median values for each treatment, with the upper and lower lines representing the first (25^th^ percentile) and third (75% quantile) quantile of the data points. N=5 for each treatment.

**
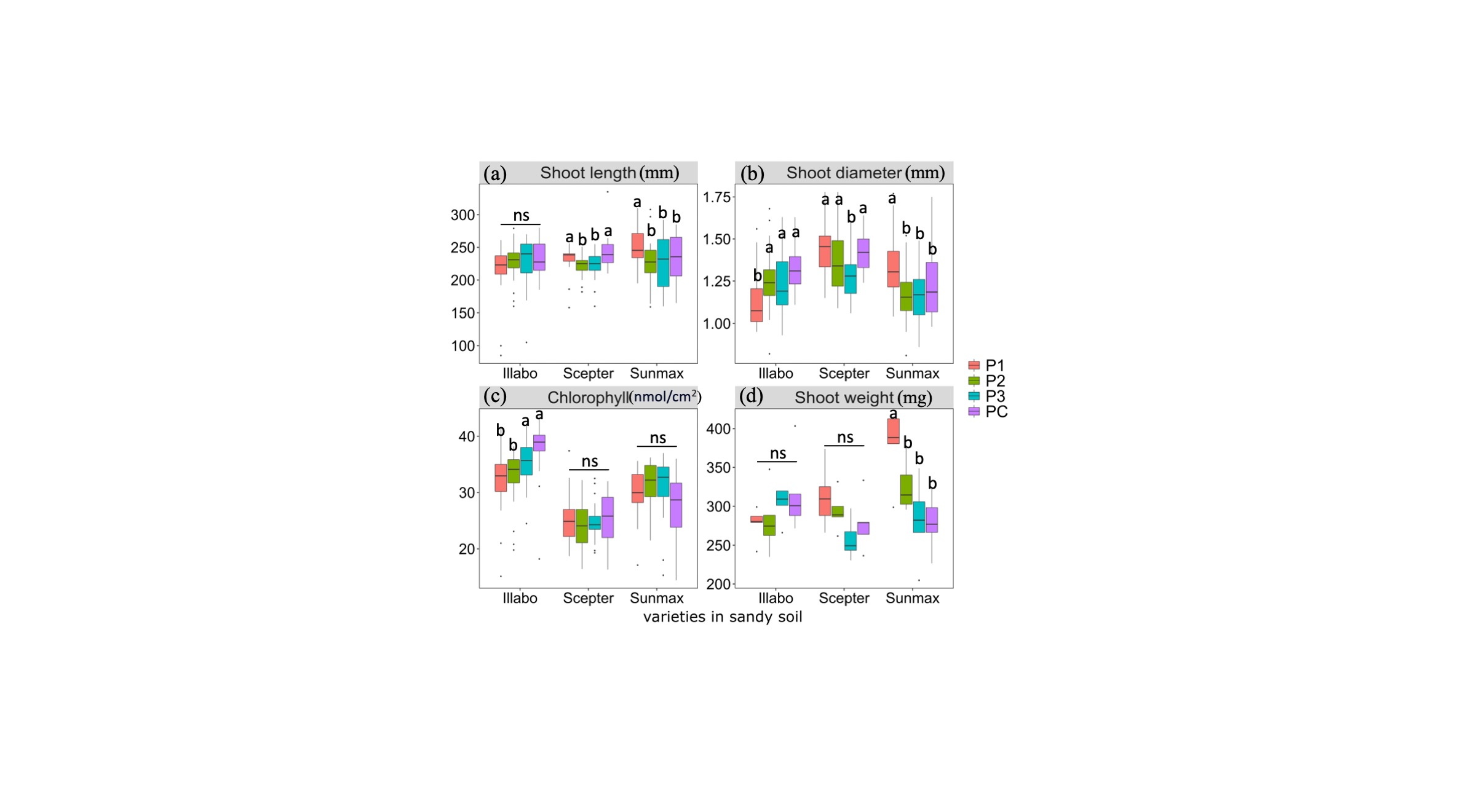
**

**Figure S3 Effects of microbial inoculant treatments on wheat phenotype after drought stress.** Figures represent (a) shoot length, (b) shoot diameter, (c) chlorophyll content, and (d) shoot weight of three wheat varieties under both drought and well-watered conditions in clay soil. In these graphs, distinct letters represent groups with significant differences, while ‘ns’ denotes no significant difference among groups. Middle back lines inserted in bars represent median values for each treatment, with the upper and lower lines representing the first (25^th^ percentile) and third (75% quantile) quantile of the data points. N=5 for each treatment.

**
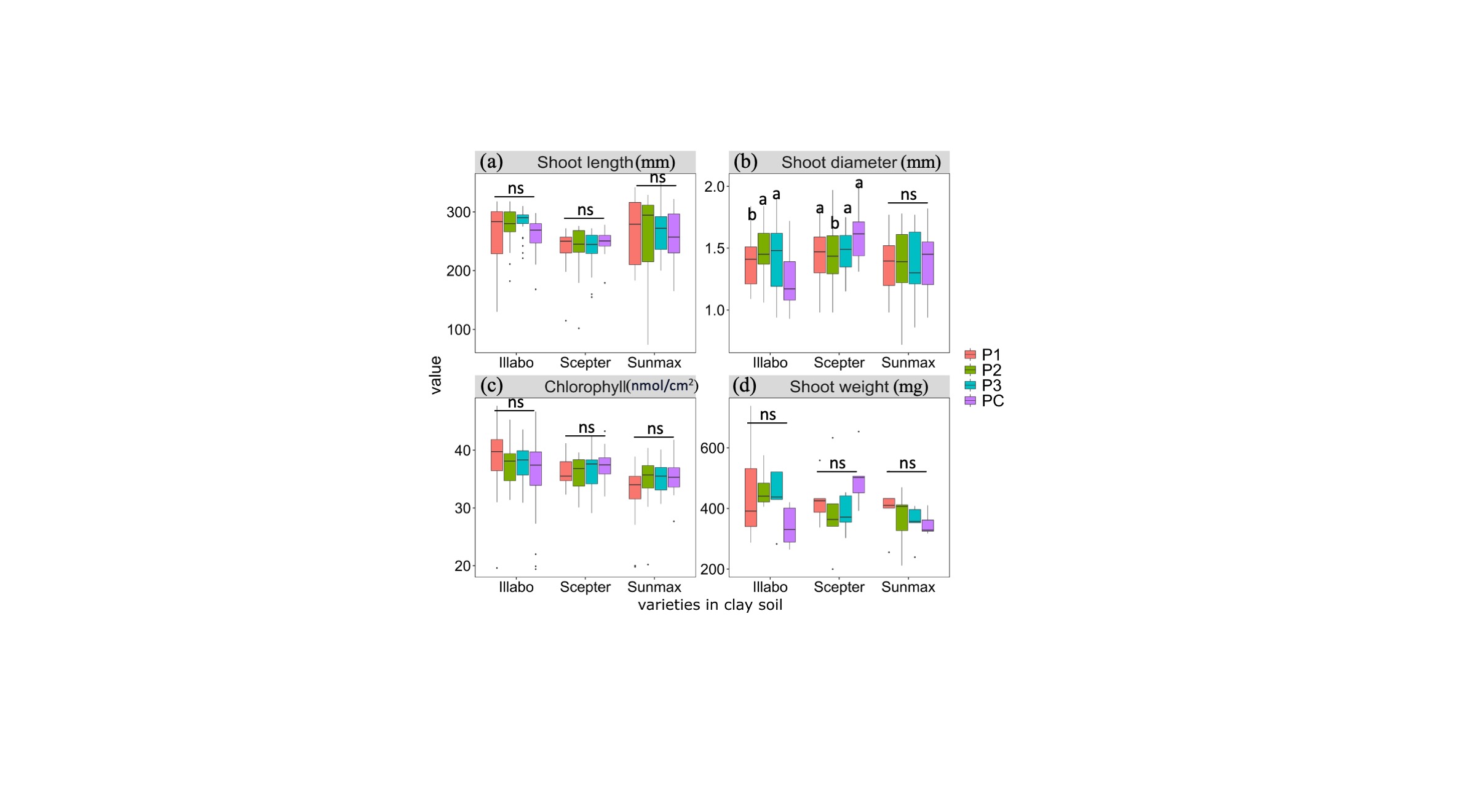
**

**Figure S4 Effects of microbial inoculants on potential soil extracellular enzyme activities after drought stress.** The graphs depict changes in (a) Carbon (C) enzyme activity, (b) Nitrogen (N) enzyme activity, and (c) Phosphorus (P) enzyme activity under both drought and well-watered conditions in clay soil. In the graphical representation, different letters indicate significance between different groups, while ‘ns’ denotes no significant differences among the groups. The middle back lines inserted in the bars represent the median values for each treatment. Furthermore, the upper and lower lines depict the first (25th percentile) and third (75% quantile) quartiles of the data points, respectively. N=5 for each treatment.

**
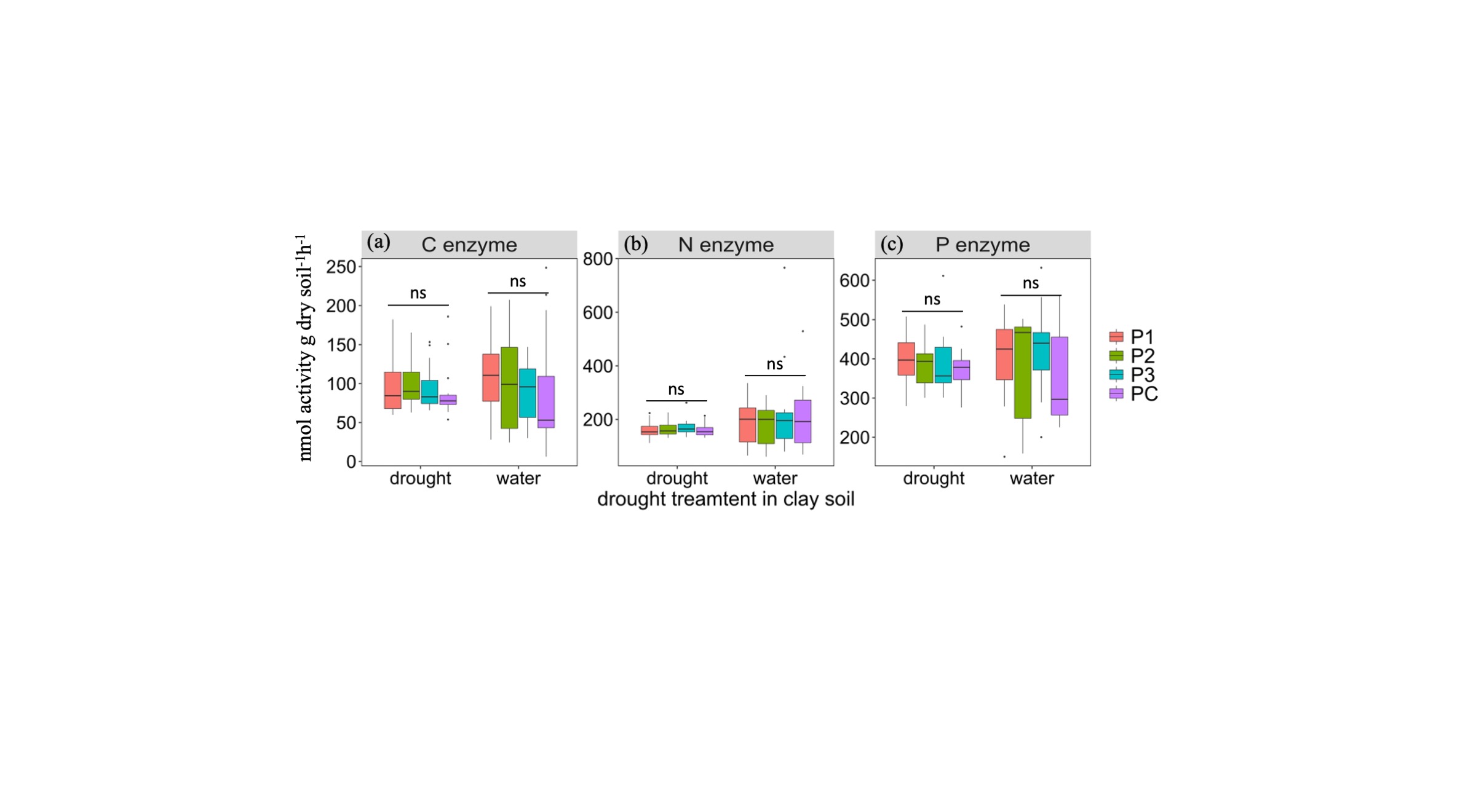
**

**Figure S5 Principal analyses (PCA) summarising the effects of microbial inoculants on the soil fungal community after drought stress.** Displayed are graphs PCA plots for fungal communities in (a) sandy bulk soil, and (b) sandy rhizosphere soil. The P-values shown on the top and bottom of the plots represent statistical significances observed in soil microbial communities between drought treatments and microbial inoculant treatments. Each grey cross within the plot represents a distinct Operational Taxonomic Unit (OTU) detected in the fungal communities of corresponding soil compartments. N=5 for each treatment.

**
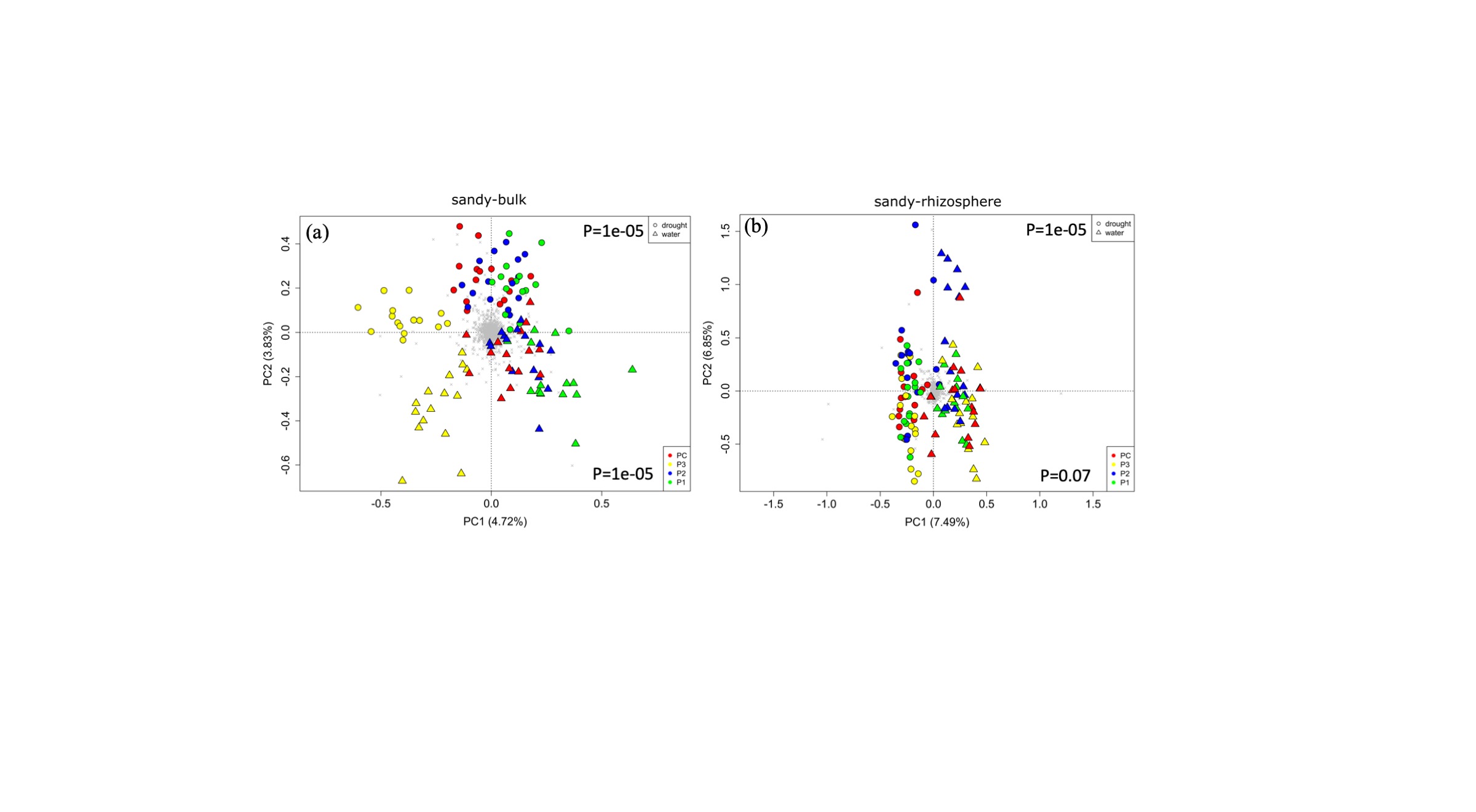
**

**Figure S6 Principal analyses (PCA) summarising the effects of microbial inoculants on the soil bacterial community after drought stress.** Displayed are graphs PCA plots for bacterial communities in (a) clay bulk soil, and (b) clay rhizosphere soil. The P-values shown on the top and bottom of the plots represent statistical significances observed in soil microbial communities between drought treatments and microbial inoculant treatments. Each grey cross within the plot represents a distinct Operational Taxonomic Unit (OTU) detected in the bacterial communities of corresponding soil compartments. N=5 for each treatment.

**
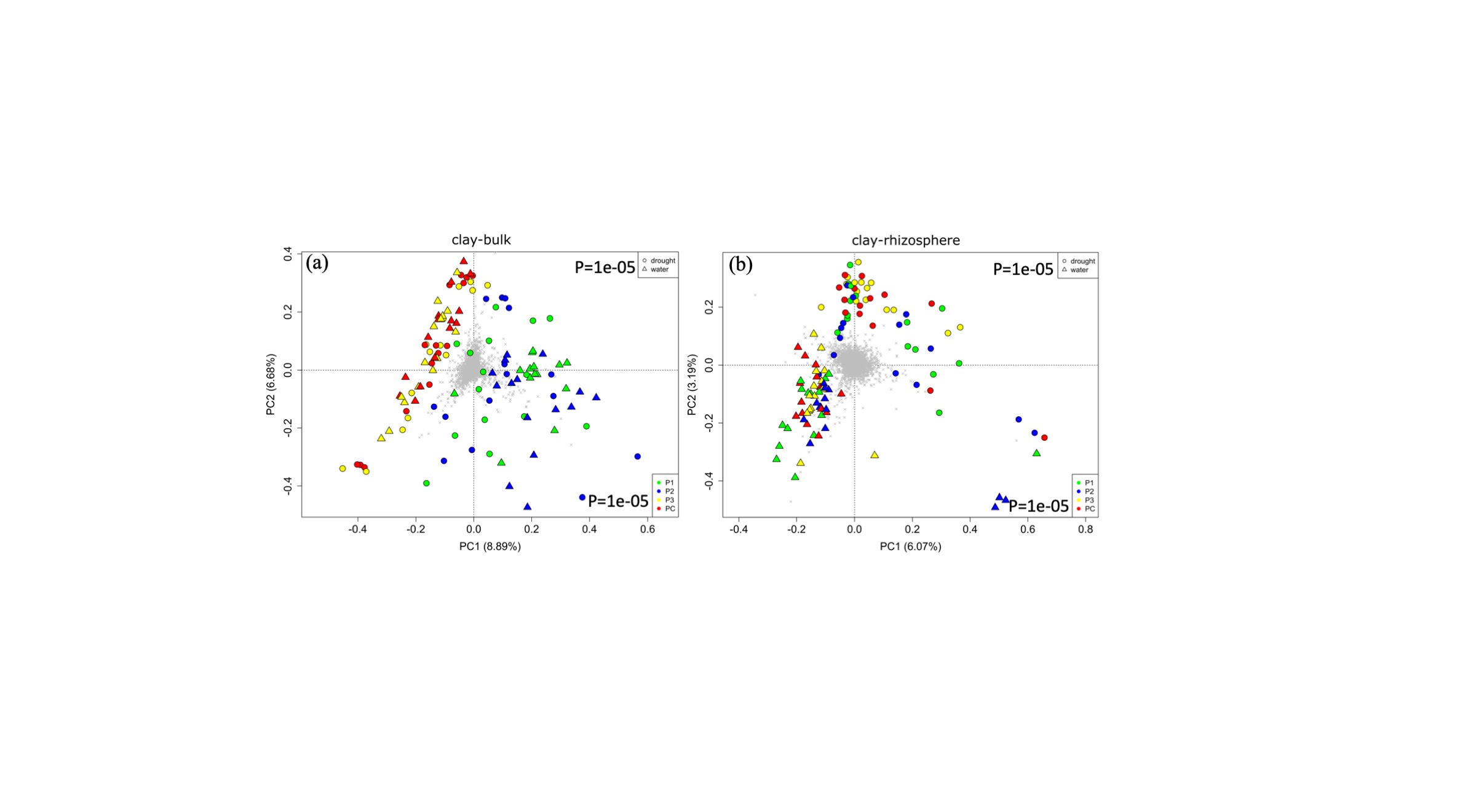
**

**Figure S7 Principal analyses (PCA) summarising the effects of microbial inoculants on the soil fungal community after drought stress.** Displayed are graphs PCA plots for fungal communities in (a) clay bulk soil, and (b) clay rhizosphere soil. The P-values shown on the top and bottom of the plots represent statistical significances observed in soil microbial communities between drought treatments and microbial inoculant treatments. Each grey cross within the plot represents a distinct Operational Taxonomic Unit (OTU) detected in the fungal communities of corresponding soil compartments. N=5 for each treatment

**
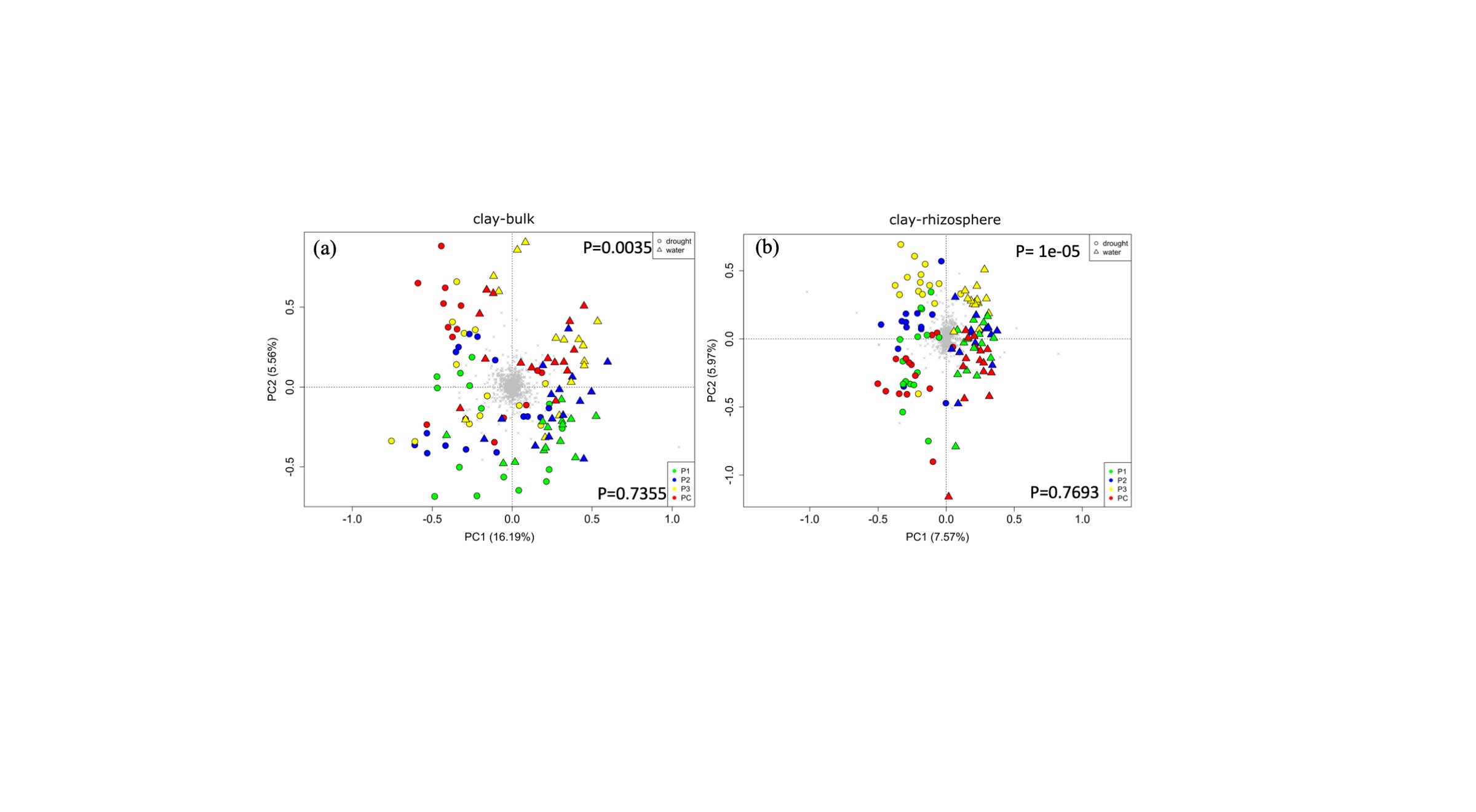
**

**Figure S8 Impacts of drought stress on soil fungal composition at genus level and biological markers under drought stress.** Two key aspects are presented the community composition of soil fungal at the genus level across different compartments, soil types, and microbial inoculant treatments.

**
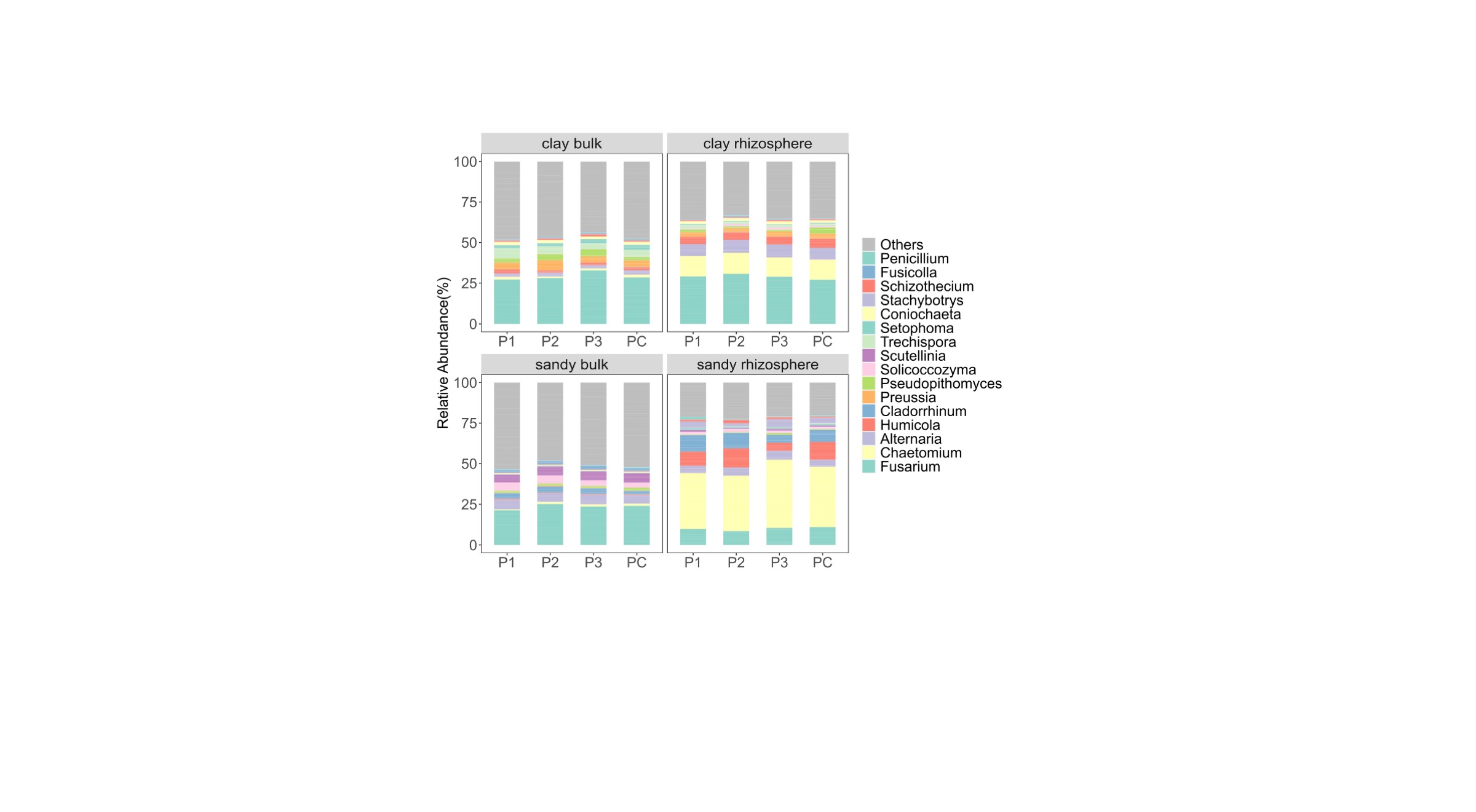
**

**
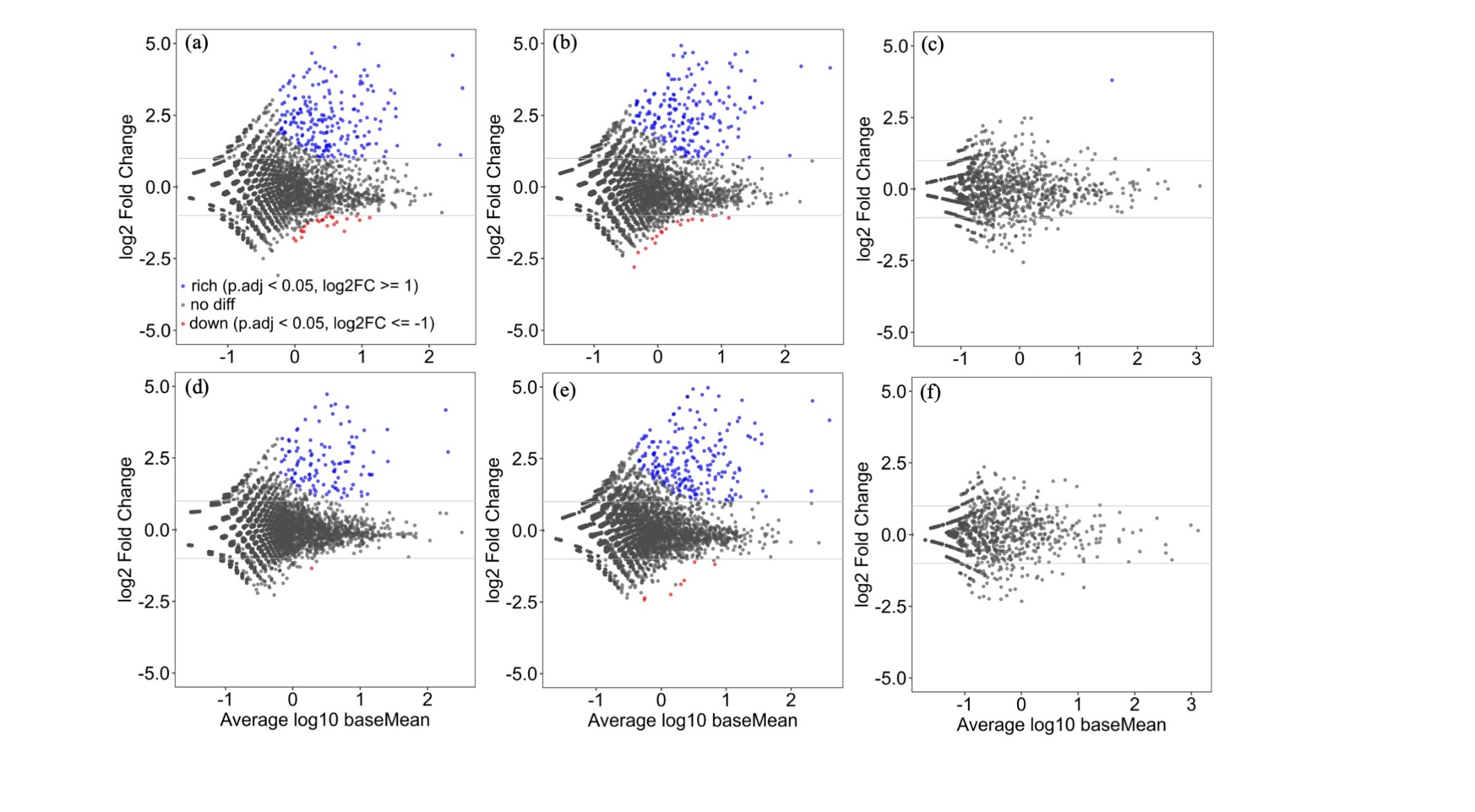
Figure S9 The number of significantly different bacterial OTUs that enriched (blue color) or decreased (red color) under drought condition** between (a) P1 and control in sandy bulk soil, (b) P2 and control in sandy bulk soil. (c) the number of significantly different fungal OTUs between P3 and control in sandy bulk soil under drought condition. The number of significantly different bacterial OTUs under drought condition between (d) P1 and control in sandy rhizosphere soil (e) P2 and control in sandy rhizosphere soil. (f) The number of significantly different fungal OTUs between P3 and control in sandy rhizosphere soil under drought condition.

**Figure S10 Network analysis presenting bacterial and fungal co-occurrence in response to different microbial inoculants in bulk and rhizosphere of sandy soil under drought condition.** This analysis was carried out after the drought stage (DS), examining the network of hub zOTUs in sandy soil under drought conditions. The network structures for each microbial inoculant treatment in bulk soil and rhizosphere soil are separately displayed: (a) P1 treatment in bulk soil; (b) P2 treatment in bulk soil; (c) P3 treatment in bulk soil; (d) PC treatment in bulk soil; (e) P1 treatment in the rhizosphere soil; (f) P2 treatment in the rhizosphere soil; (g) P3 treatment in the rhizosphere soil; and (h) PC treatment in the rhizosphere soil. In these networks, circles of the same colors represent the same taxa. BOtu refers to bacteria, and **
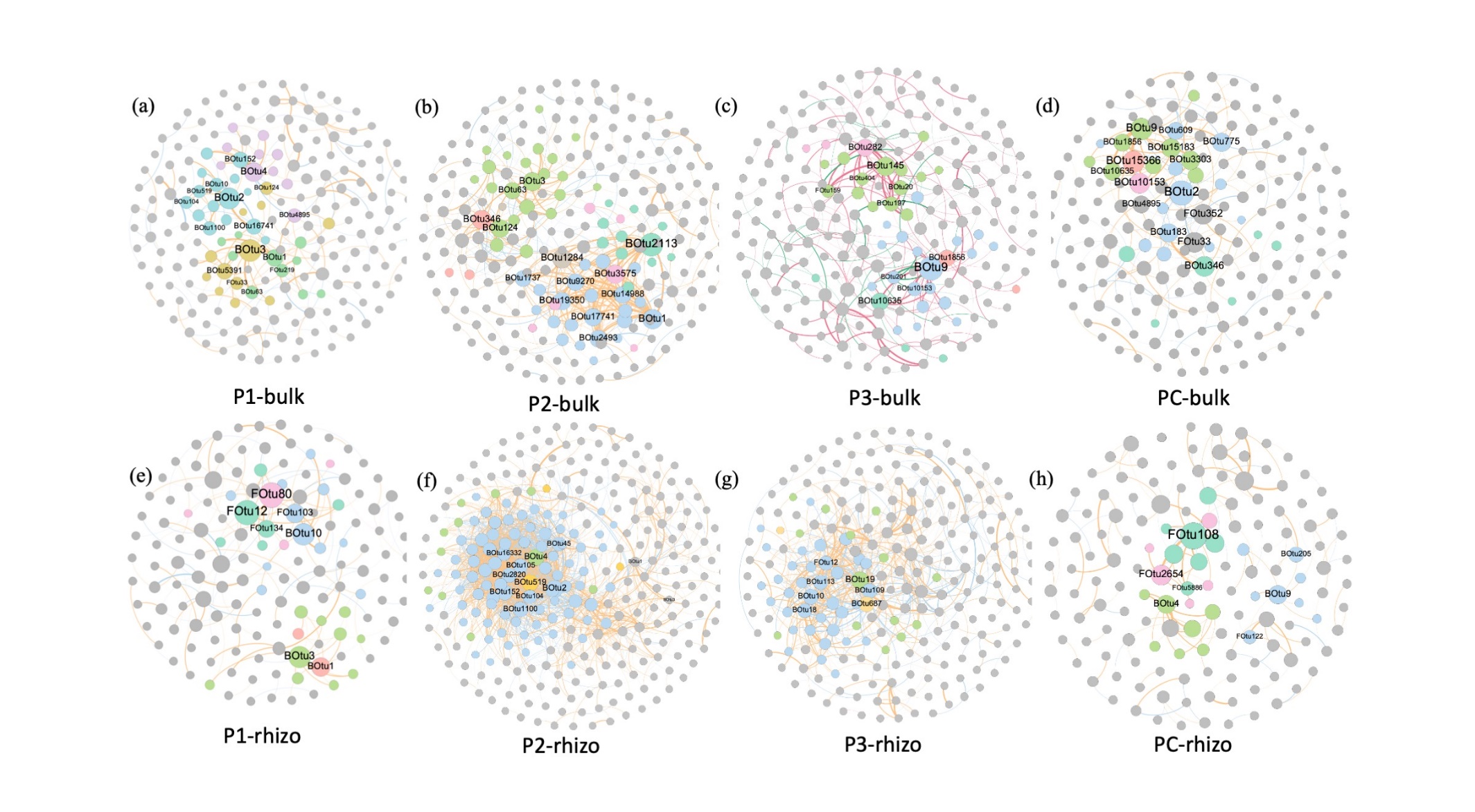
**FOtu refers to fungi.

**
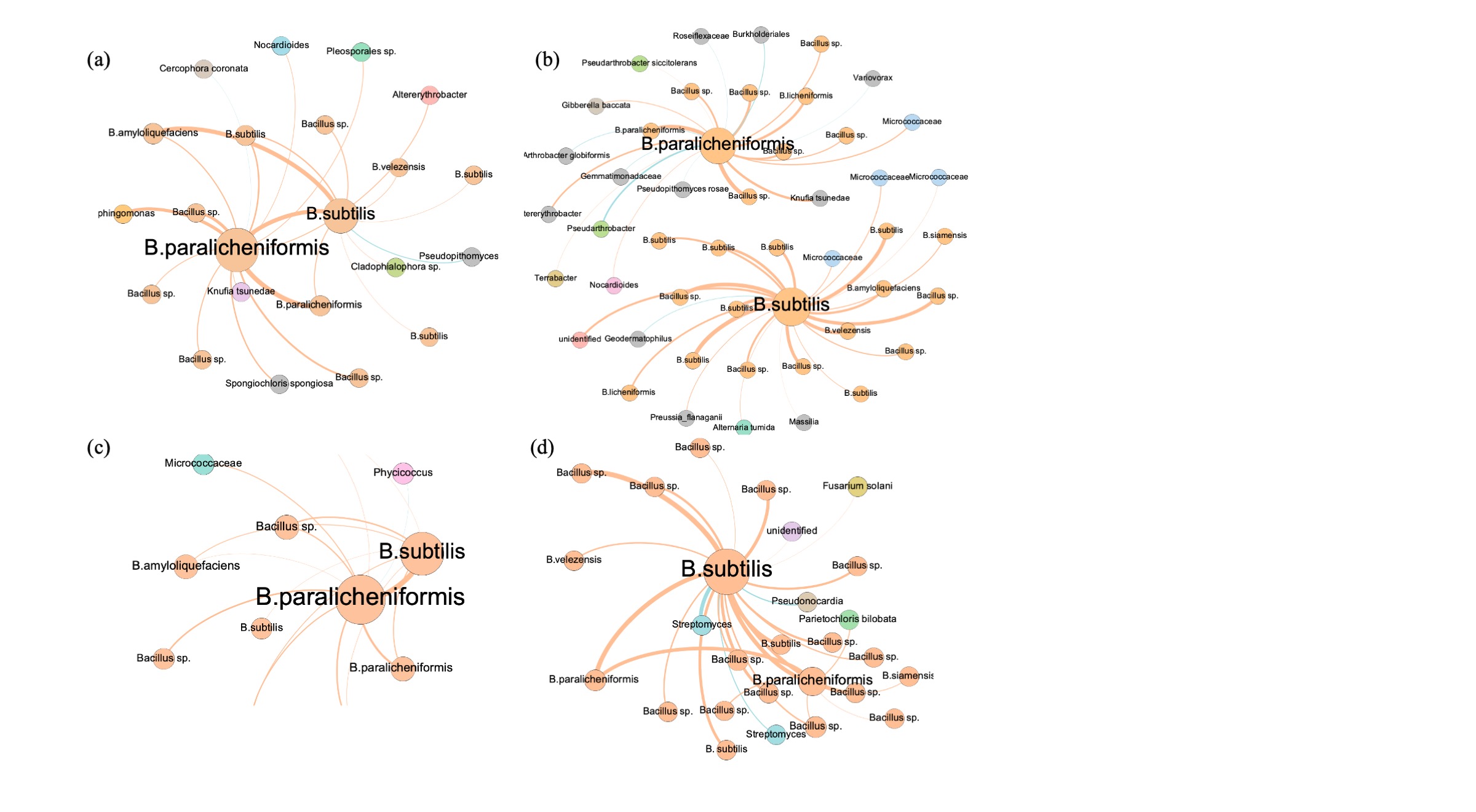
Figure S11 Network analysis revealed the co-occurrence of OTU1 and OTU3 with other species under drought conditions.** Specifically (a) with P1 treatment in sandy bulk soil, (b) with P2 treatment in sandy bulk soil, (c) with P1 treatment in sandy rhizosphere soil and (d) with P2 treatment in sandy rhizosphere soil.
