# Supplementary material for: Microbial inoculants with higher capacity to colonize soils improved wheat drought tolerance
Source: Microb Biotechnol. 2023 Oct 10;16(11):2131–44. doi: 10.1111/1751-7915.14350 (PMC10616649; doi:10.1111/1751-7915.14350)
Supplement: Supplementary file 5 — Table S4 [file MBT2-16-2131-s003.docx]

| Factors | Df | SumsOfSqs | MeanSqs | F.Model | R2 | Pr(>F) |  |
| --- | --- | --- | --- | --- | --- | --- | --- |
| Soil types | 1 | 54.273 | 54.273 | 209.44 | 0.30689 | 1.00E-05 | *** |
| Compartments | 1 | 9.358 | 9.3579 | 26.427 | 0.05292 | 1.00E-05 | *** |
| Drought treatments | 1 | 3.192 | 3.1924 | 8.6954 | 0.01805 | 1.00E-05 | *** |
| Microbial inoculants | 3 | 6.93 | 2.31007 | 6.4034 | 0.03919 | 1.00E-05 | *** |
| Varieties | 2 | 0.553 | 0.2764 | 0.74002 | 0.00313 | 0.7892 |  |

**Table S4**

| Factors | Df | SumsOfSqs | MeanSqs | F.Model | R2 | Pr(>F) |  |
| --- | --- | --- | --- | --- | --- | --- | --- |
| Soil types | 1 | 64.923 | 64.923 | 207.33 | 0.3052 | 1.00E-05 | *** |
| Compartments | 1 | 16.603 | 16.6030 | 39.958 | 0.07805 | 1.00E-05 | *** |
| Drought treatments | 1 | 4.205 | 4.2046 | 9.5176 | 0.01977 | 1.00E-05 | *** |
| Microbial inoculants | 3 | 0.858 | 0.28584 | 0.63411 | 0.00403 | 0.9644 |  |
| Varieties | 2 | 0.689 | 0.34445 | 0.76514 | 0.00324 | 0.7357 |  |

**Table S5**
